# Haplotype-resolved genomes of two buckwheat crops provide insights into their contrasted rutin concentrations and reproductive systems

**DOI:** 10.1186/s12915-023-01587-1

**Published:** 2023-04-17

**Authors:** Hao Lin, Yingjun Yao, Pengchuan Sun, Landi Feng, Shuo Wang, Yumeng Ren, Xi Yu, Zhengxiang Xi, Jianquan Liu

**Affiliations:** 1 Key Laboratory for Bio-Resource and Eco-Environment of Ministry of Education & Sichuan Zoige Alpine Wetland Ecosystem National Observation and Research Station, College of Life Science, Sichuan University, Chengdu, China; 2 State Key Laboratory of Dao-Di Herbs, Beijng, 100700 People’s Republic of China; 3 State Key Laboratory of Herbage Improvement and Grassland Agro‐Ecosystems, College of Ecology, Lanzhou University, Lanzhou, 730000 China

**Keywords:** Buckwheat, Haplotype-resolved genome, Rutin concentrations, Reproductive systems

## Abstract

**Background:**

Two widely cultivated annual buckwheat crops, *Fagopyrum esculentum* and *F. tataricum*, differ from each other in both rutin concentration and reproductive system. However, the underlying genetic mechanisms remain poorly elucidated.

**Results:**

Here, we report the first haplotype-resolved chromosome-level genome assemblies of the two species. Two haplotype genomes of *F. esculentum* were assembled as 1.23 and 1.19 Gb with N50 = 9.8 and 12.4 Mb, respectively; the two haplotype genomes of *F. tataricum* were 453.7 and 446.2 Mb with N50 = 50 and 30 Mb, respectively. We further annotated protein-coding genes of each haplotype genome based on available gene sets and 48 newly sequenced transcriptomes. We found that more repetitive sequences, especially expansion of long terminal repeat retrotransposons (LTR-RTs), contributed to the large genome size of *F. esculentum*. Based on the well-annotated sequences, gene expressions, and luciferase experiments, we identified the sequence mutations of the promoter regions of two key genes that are likely to have greatly contributed to the high rutin concentration and selfing reproduction in *F. tartaricum*.

**Conclusions:**

Our results highlight the importance of high-quality genomes to identify genetic mutations underlying phenotypic differences between closely related species. *F. tataricum* may have been experienced stronger selection than *F. esculentum* through choosing these two non-coding alleles for the desired cultivation traits. These findings further suggest that genetic manipulation of the non-coding promoter regions could be widely employed for breeding buckwheat and other crops.

**Supplementary Information:**

The online version contains supplementary material available at 10.1186/s12915-023-01587-1.

## Background

Two species of buckwheat, *Fagopyrum esculentum* and *F. tataricum* (Polgonaceae), are widely cultivated, important, traditional crops [1]. These two pseudo-cereals contain nutritious proteins, lipids, dietary fiber, minerals, and flavonoids but no gluten [2] and are thus healthy and functional foods especially for individuals with gluten intolerance [3]. However, the two differ from each other with respect to two biological traits. First, Rutin concentration is higher in *F. tataricum* than in *F. esculentum *[4]. Rutin, an important flavonoid with a bitter taste, has antioxidant properties [5] and is associated with reducing hypertension, hyperlipidemia, and hyperglycemia [6]. Rutin synthesis is evolutionarily conserved in plants [7, 8] and this has been confirmed in *F. tataricum* [1]. Second, *F. esculentum* is completely outcrossing and depends on insects for pollination, while self-compatible *F. tataricum* can set seed without pollinators. *F. esculentum* is self-incompatible (SI) because of the tightly linked S genes at the S locus, as in other plants [9–11]. Numerous self-compatible plants with this ancestral determinant mechanism are the result of mutations and reduced gene expressions in key genes on the S-determining cluster [12, 13]. This switching phenomenon tends to occur in domesticated species, suggesting that there may be a cost associated with this route in natural populations, perhaps because the *S-RNase* has a function outside self-incompatibility [14]. These two characters are critical for selection of both sites and uses when cultivating the two crops. It would be interesting to know the underlying genetic variations that lead to these phenotypic differences between the two closely related species.

A comprehensive comparison of genome sequences may provide a basis for our understanding of the development of these differences. Draft genomes of both *F. tataricum* and *F. esculentum* have been reported [1, 15]. However, the genes relevant to both rutin synthesis and self-incompatibility are difficult to be accurately aligned, annotated, and compared. In particular, the high heterozygosity and high cconentration of repeated sequences of *F. esculentum* hinder the assembly of a high-quality genome based on second-generation sequencing data [15]. Here, we report the first haplotype-resolved chromosome-level genome assemblies of both *F. esculentum* and *F. tataricum*, relying on HiFi sequencing and Hi-C scaffold technology. Based on these two high-quality genomes, we found down-regulated expression of one key gene in the rutin synthesis pathway in *F. esculentum*, which may have resulted in the limited rutin synthesis, thereby reducing the overall rutin content in *F. esculentum*. In addition, we also found that self-compatibility of *F. tataricum* resulted from the inhibition of *S-RNase* expression at the S locus. We believe that the highly improved genome data for both buckwheat species presented in the current study will provide important resources for future genetic breeding and will help to deepen our understanding of biology and evolution of these two crops and congeners in the family Polygonaceae.

## Results

### *De novo* genome assembly of *F. esculentum* and *F. tataricum*

**Fig. 1:**
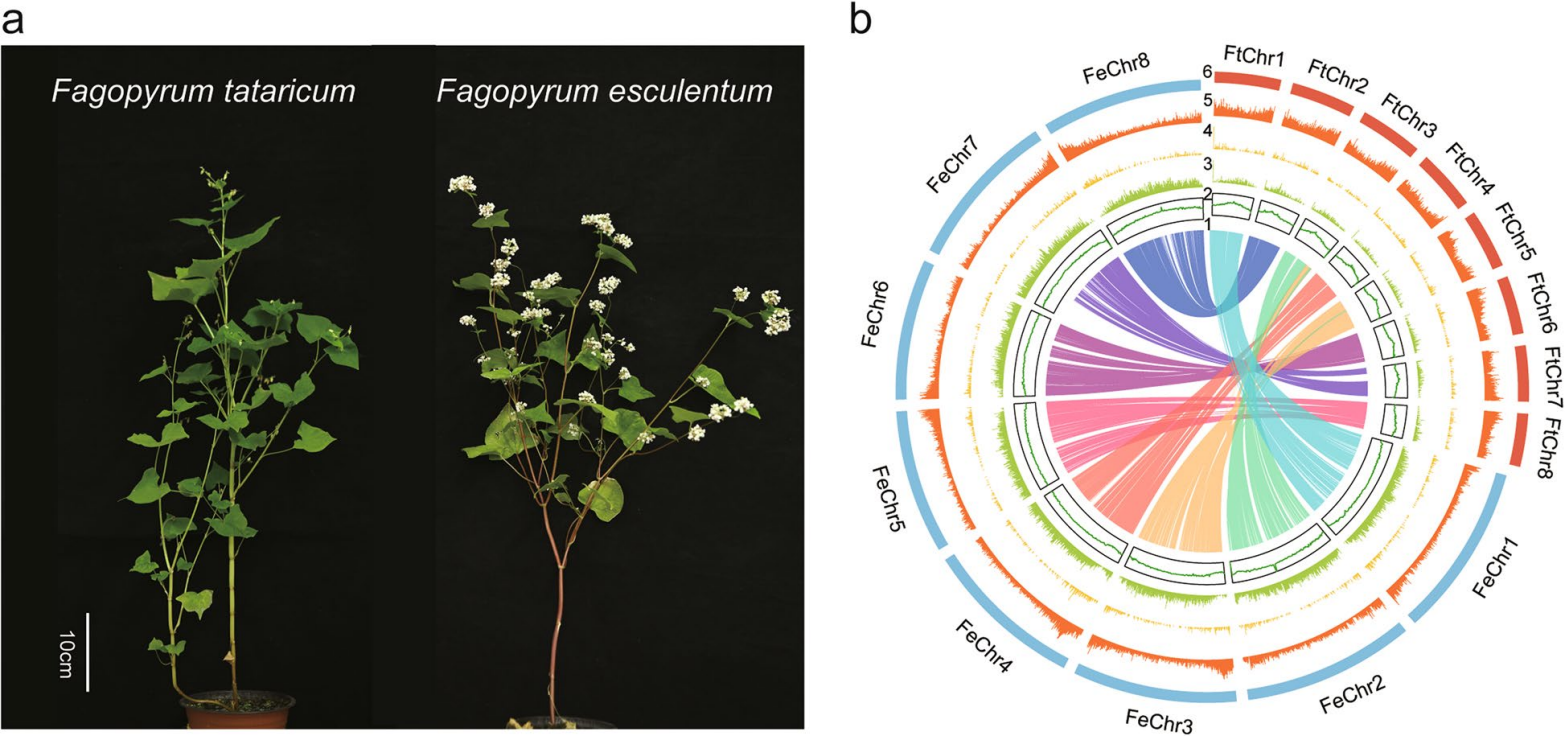
Genome assemblies of *F. esculentum* (Fe) and *F. tataricum* (Ft). **a** Photograph of whole plants of *F. tataricum* cultivar “Qianku3” and *F. esculentum* cultivar “Xinong9976”. **b** Circos plot showing the genomic features of the two buckwheat species. The features from inside to outside are (1) inter-genome collinear blocks connected by curved lines, (2) GC content, (3) Gypsy density, (4) Copia density, (5) gene density, and (6) chromosome number. All distributions are drawn in a window size of 200 kb

We used *F. esculentum* cv. Xinong9976 (Xinong9976) and *F. tataricum* cv. Qianku3 (Qianku3) for genome sequencing and assembly (Fig. 1a). Before the de novo assembly of the Xinong9976 and Qianku3, the genome size was estimated using the k-mer distribution assessment (*k* = 17) from Illumina short reads. The genome size of Xinong9976 is about 1.27 Gb and Qianku3 is about 579.36 Mb. Based on the HiFi sequencing method from Pacific Biosciences (PacBio), we obtained 37.05 Gb of PacBio long reads (Additional file 3: Table S1 and Additional file 1: Fig. S1), and a “Xinong9976” monoploid assembly resulted in 1.38 Gb with high contiguity (Additional file 1: Fig. S2). A total of 798 contigs with an N50 of 36.4 Mb were recovered and the longest was approximately 72.89 Mb. The “Xinong9976” sequences were further phased into two haplotypes: “Fe-haplotype 1” and “Fe-haplotype 2” (Table 1). Using the Hi-C data, the two haplotype genomes were anchored onto eight pseudo-chromosomes (Fig. 1b, Additional file 1: Fig. S3 and Fig. S4), respectively. The “Fe-haplotype 1” genome assembly had a total size of 1.23 Gb, including 282 contigs with N50 = 9.8 Mb and the largest contig size was 44.1 Mb (Table 1). The “Fe-haplotype 2” genome assembly had a total size of 1.19 Gb, including 246 contigs with N50 = 12.4 Mb and the largest contig size was 52.07 Mb (Table 1). The genome Benchmarking Universal Single-Copy Orthologs (BUSCO) analysis against the embryophyte odb10 database detected 95.1% of complete BUSCOs, within the genome assembly of “Fe-haplotype 1,” and found 92.6% of complete BUSCOs, within the genome assembly of “Fe-haplotype 2” (Additional file 3: Table S3).

**Table 1:** Statistics of the assembly of the *F. esculentum* and *F. tataricum* genome

	Fe-haplotype 1	Fe-haplotype 2	Ft-haplotype 1	Ft-haplotype 2
Assembly size (bp)	1,234,282,574	1,193,797,812	453,711,210	446,177,767
Number of contigs	282	246	26	90
Max. contig length (bp)	44,131,638	52,069,794	60,977,960	60,914,563
Min. contig length (bp)	16,930	19,085	43,541	19,262
Contig N50 length (bp)	9,830,627	12,384,143	49,984,048	30,040,728
Contig N50 count	34	28	5	6
Contig N90 length (bp)	2,532,085	2,352,370	22,685,761	5,507,589
Contig N90 count	122	108	9	16
Gap content (%)	0.01	0.01	< 0.01	0.01
GC content (%)	40.50	40.10	38.27	38.23
Gene number	49,546	49,080	36,778	34,509
Repeat content (%)	62.58	61.88	48.54	47.39

*F. tataricum* cv. Qianku3 was sequenced using the PacBio method, resulting in a genome coverage of 64.6 × and further assembled with hifiasm (Additional file 3: Table S2, Additional file 1: Fig. S5 and Fig. S6). We generated a 528.63 Mb monoploid assembly with a contig N50 of 50.06 Mb for Qianku3. It was then phased into two haplotypes, “Ft-haplotype 1” and “Ft-haplotype 2” (Table 1, Additional file 1: Fig. S7 and Fig. S8). Using Hi-C data from the published datasets, approximately 98.11% of sequences were anchored onto pseudo-chromosomes in the two haplotypes (Fig. 1b). The genome size of the final assembly for “Ft-haplotype 1” was 453.7 Mb with 26 contigs (N50 = 50.0 Mb) (Table 1). The genome size of “Ft-haplotype 2” was 446.2 Mb with 90 contigs (N50 = 30.0 Mb) (Table 1). The quality of the assembly was evaluated using the BUSCO. The results showed that the completeness of Ft-haplotype 1 is 96.6%, and the completeness of Ft-haplotype 2 is 96.3% (Additional file 3: Table S3). The haplotyped genome assembly of *F. tataricum* has good genomic collinearity with previously published *F. tataricum* cv. Pinku1 genome assembly (Additional file 1: Fig. S9 and Fig. S10).

### Repeat and gene annotations

**Fig. 2:**
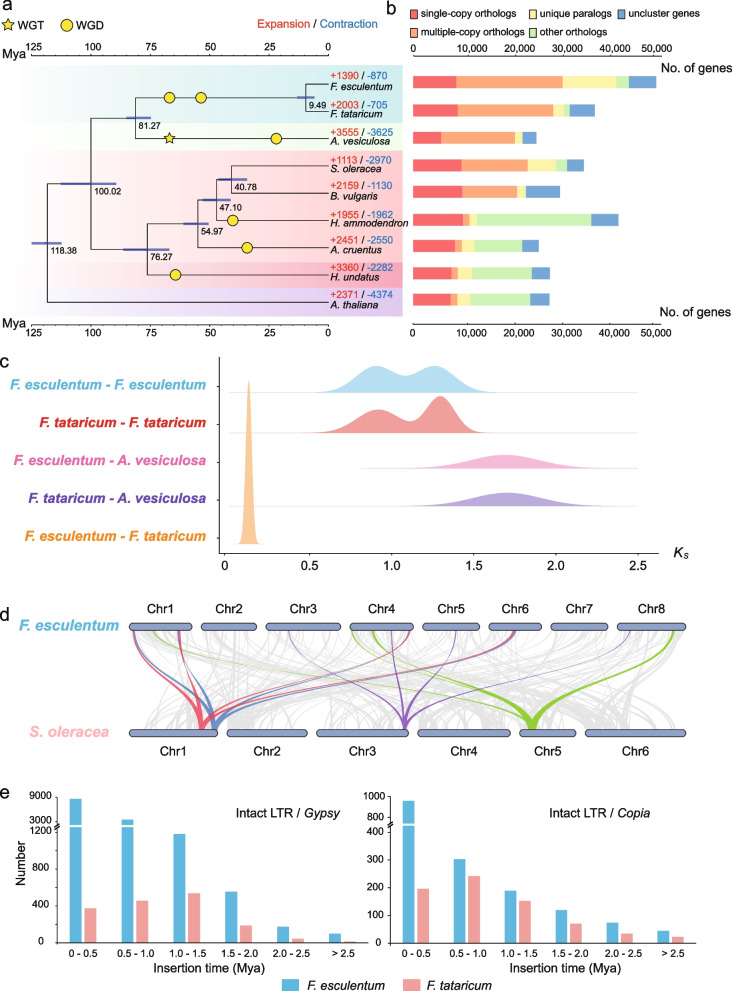
Evolution of the Buckwheat Genomes. **a** Phylogenetic tree showing evolutionary relationships between the two buckwheat species and seven other plants, including their divergence time and whole-genome duplications that occurred within the lineage. All branch bootstrap values are 100. The estimated divergence time (million years ago, Mya) is indicated at each node; bars are 95% confidence intervals. **b** Genes of *F. tataricum*, *F. esculentum*, and other sequenced genomes are divided into five classes. Gene family expansions are indicated in red, and gene family contractions in blue. **c** *Ks* values revealed two WGD events shared by *F. tataricum* and *F. esculentum*. **d** Collinear relationship between *F. esculentum* and *S. oleracea* chromosomes. The collinearity pattern shows that typically an ancestral region in *S. oleracea* genome can be traced to four regions in *F. esculentum*. Gray bands in the background indicate large syntenic blocks between the genomes, some of the 1:4 blocks are highlighted in different colors. **e** The number of intact LTR-RTs in the *F. tataricum* and *F. esculentum* genomes

We identified 790.8 Mb (62.6%) of repetitive sequences in *F. esculentum* (Fe-haplotype 1), including 45.1% of retrotransposons and 1.4% of DNA transposons (Supplemental Table 4). LTR-RTs were found to account for 43.0% of the genome (Additional file 3: Table S4). A very recent LTR-RTs burst event was detected in the *F. esculentum* genome, dating back to 0.3–0.5 million years ago (Mya), based on the divergence of the terminal sequences of the repeats (Fig. 2e). *F. tataricum* (Ft-haplotype 1) comprises a small ratio of repeat sequences (255.3 Mb, 48.5% of genome), including 20.2% of retrotransposons and 2.5% of DNA transposons (Additional file 3: Table S4). LTR-RTs were found to account for 16.5% of the *F. tataricum* genome (Additional file 3: Table S4). Compared with *F. tataricum*, the large-scale expansion of LTR-RTs in *F. esculentum* may be one of the important reasons for its large genome size.

We masked the repeat regions and annotated the four genomes using a comprehensive strategy including all newly available RNA-seq data (Additional file 3: Table S5). For *F. esculentum*, 49,546 protein-coding genes were identified in the Fe-haplotype 1 with an average gene length of 2893 bp, whereas 49,080 protein-coding genes were identified in Fe-haplotype 2 with an average gene length of 2865 bp (Table 1 and Additional file 3: Table S6). Two haplotype genome annotation showed over 96.6% and 94.1% coverage of the BUSCO set of orthologs (Additional file 3: Table S7). For *F. tataricum*, a total of 36,778 and 34,509 protein-coding genes were predicted for the two haplotype genomes: Ft-haplotype 1 and Ft-haplotype 2, respectively (Additional file 3: Table S8), and 66,385 (93.12%) of these genes were located on the assembled chromosomes. The BUSCO of genome annotation is 97.5% and 96.9% respected for Ft-haplotype 1 and Ft-haplotype 2 (Additional file 3: Table S7). Furthermore, in the two diploid-resolved genomes, 93.1% and 94.8% predicted genes were successfully annotated by at least one functional database (Additional file 3: Table S9 and Table S10), indicating near completion of both assemblies and annotations.

### Phylogenetic and whole-genome duplication analyses

To investigate genome evolution, we compared Ft-haplotype 1 and Fe-haplotype 1 to seven other plant species, using *A. thaliana* as an outgroup (Additional file 3: Table S11). We used 986 single-copy gene families among the nine species to construct a maximum likelihood phylogenetic tree, which showed that *F. esculentum* was sister to *F. tataricum*. Polygonaceae (*F. esculentum*, and *F. tataricum*) had a close relationship with Droseraceae (*A. vesiculosa*) followed by Amaranthacea (*S. oleracea*, *B. vulgaris*, *H. ammodendron*, and *A. cruentus*), whilst Cactaceae (*H. undatus*) was distant from the other plants (Fig. 2a). We found that *F. esculentum* and *F. tataricum* diverged from the common ancestor about 9.49 million years ago (Mya), and Polygonaceae separated from Droseraceae around 81.27 Mya (Fig. 2a). The well-supported phylogeny was largely congruent with previous phylogenetic analyses, and it was used as a framework for further comparative and evolutionary genomic analyses.

We further investigated whole-genome duplication (WGD) events. We used the distribution of synonymous substitution rates (*Ks*) per gene between collinear paralogous genes to identify WGD events. We identified a total of 1331 syntenic blocks, containing 16,692 paralogous gene pairs that accounted for 33.69% of the predicted *F. esculentum* genes (Additional file 3: Table S12), while 1010 syntenic blocks, containing 11,730 paralogous gene pairs were identified in *F. tataricum* (Additional file 3: Table S13). The large paralogous regions identified in the intra-genome comparison of *F. esculentum* and *F. tataricum* suggested two whole genome duplication events (Fig. 2c). The *Ks* distribution of these duplicated gene pairs peaked at 0.90 and 1.26 (Fig. 2c). Based on the *Ks* peak of the orthologous gene pairs between *F. esculentum* and *F. tataricum*, we inferred that ancestral species of the two buckwheats experienced two WGD events. Synteny analyses comparing the genomes of *F. esculentum* and *S. oleracea* also showed clear evidence of two WGD events in the genus *Fagopyrum* (Fig. 2d). For each genomic region in *S. oleracea*, we found four matching regions in *F. esculentum* with a similar level of divergence (Fig. 2d). The genome of *S. oleracea* had not experienced any recent WGD after the hexaploidization event shared by core eudicots. The overall 4:1 syntenic relationship between *F. esculentum* and *S. oleracea* suggested that the genus *Fagopyrum* experienced two WGD events after its divergence from *S. oleracea* (Additional file 2: Fig. S11 and Fig. S12).

### Gene family analyses

A total of 45,233 genes (91.30% of the 49,546 ones in the Fe-haplotype genome) in *F. esculentum* clustered into 16,984 gene families, which included 7697 (45.32%) gene families shared by all nine species and 1484 (8.74%) families that were specific to *F. esculentum* (Fig. 2b and Additional file 3: Table S14). The 36,778 protein coding genes in the *F. tataricum* Ft-haplotype genome were grouped into 16,001 gene families, and 609 gene families were *tataricum*-specific. (Fig. 2b and Additional file 3: Table S14). In addition, we found 2268 gene families (containing 10,572 genes) that appeared to be unique to *Fagopyrum*. These lineage-specific gene families in *Fagopyrum* were significantly enriched in various biosynthetic categories (e.g., protein ubiquitination, defense response and regulation of transcription) and stress-related categories (Additional file 3: Table S15).

Analyses of gene family expansion and contraction revealed 1390 gene families that have undergone expansion in *F. esculentum*, whereas 870 gene families became smaller (Additional file 3: Table S16). In the *F. tataricum* genome, 2003 gene families were expanded, and 705 gene families contracted (Additional file 3: Table S16). These expanded gene families were then annotated using GO terms. The *F. tataricum* GO annotations were mainly related to flavonoid biosynthetic process, defense response, and cell surface receptor signaling pathway in the “biological process” term (Additional file 3: Table S17 and Additional file 2: Fig. S13). The *F. esculentum* expanded gene families’ GO annotations were mainly related to rejection of self-pollen, DNA repair, and DNA recombination (Additional file 3: Table S18 and Additional file 2: Fig. S14).

### Rutin biosynthesis

**Fig. 3:**
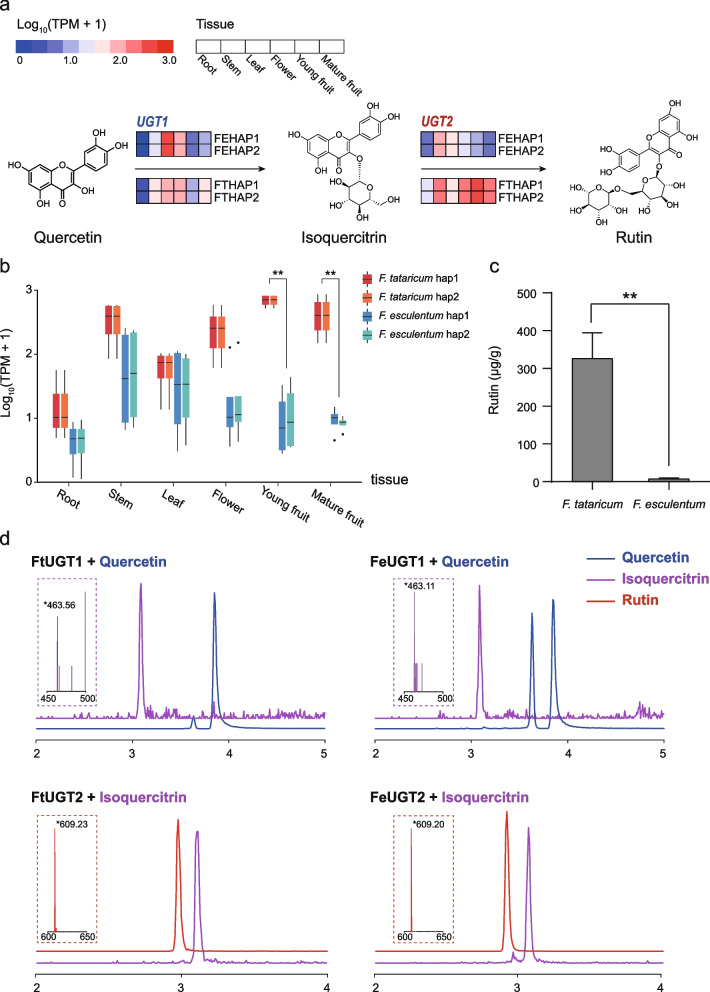
The Genes Involved in Rutin Biosynthesis. **a** A simplified representation of the flavonoid biosynthetic pathway (adapted from the KEGG PATHWAY Database: http://www.genome.jp/kegg/pathway.html) and the key enzyme-coding genes for each step. The expression value of each gene is indicated by the different colors representing log_10_(TPM + 1) in six tissues: root, stem, leaf, flower, young fruit, and mature fruit. **b** Different expression profiles of the gene *UGT2* across six tissues of *F. tataricum* and *F. esculentum* (significance was tested by paired *t* test **P-value < 0.01). **c** Histogram of rutin content in mature fruit of *F. tataricum* and *F. esculentum* (significance was tested by paired *t* test **P-value < 0.01). **d** LC–ESI–MS/MS analysis of four functional UGT genes. In catalytic reactions, FtUGT1 and FeUGT1 use quercetin and UDP-glucose as substrates, FtUGT2 and FeUGT2 use isoquercitrin and UDP-rhamnose as substrates to generate corresponding flavonoids compounds. The characteristic mass spectrum peaks of products from each reaction are displayed in the dashed box of each track

Rutin (quercetin-3-rutinoside) is a flavonoid synthesized in higher plants as a reducing agent against UV radiation and diseases [16]. The two buckwheat crops differ from each other with respect to their concentrations of rutin [17]. The large majority of common buckwheat presented rutin contents between the range from 25 mg/100 g DW to 15 mg/100 g DW. The rutin content of tartary buckwheat, ranging from 1193 mg/100 g DW to 979 mg/100 g DW of, was decidedly higher than that of common buckwheat [18]. The rutin content in *F. tataricum* was significantly higher than that in *F. esculentum* within mature fruit profiled by HPLC (Fig. 3c). Using comparative analysis of gene homologs of four haplotype genomes related to rutin synthesis in *F. tataricum* [1], we identified all homologous genes in *F. esculentum* that encode six enzymes in the rutin biosynthetic pathway. In addition, we also identified key protein-encoding genes responsible for the conversion of quercetin to isoquercetin (quercetin 3-O-glucoside) and ultimately rutin synthesis in *F. esculentum* by homology comparison with two genes *FtUGT73BE5* (named as *UGT1*) and *FtUGT79A15* (named as *UGT2*) of *F. tataricum* [1]. Based on the transcriptomes of different tissues of the two species, we found that the upstream genes of the rutin synthesis pathway showed no distinct expression difference (Fig. 3a and Additional file 2: Fig. S15).

**Fig. 4:**
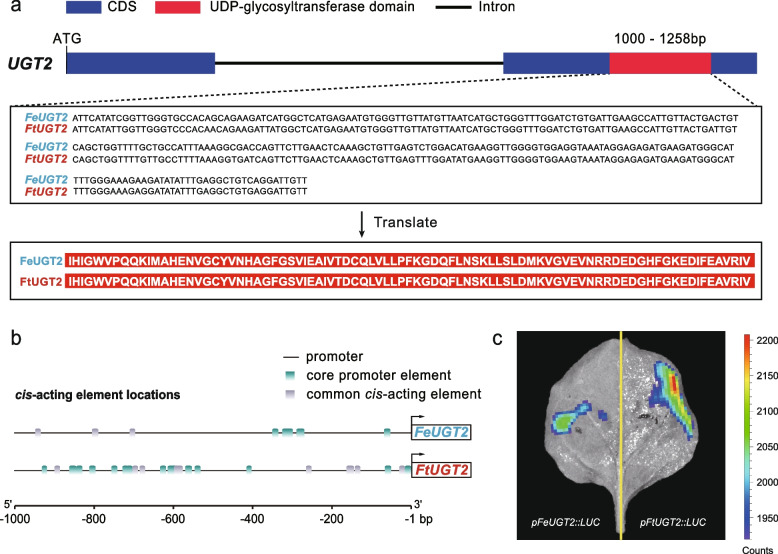
The gene structure and binding activities of *UGT2* genes of two species. **a** Gene structure of *UGT2* in *F. esculentum* and *F. tataricum*. Blue square represents CDS. Red square represents UDP-glycosyltransferase domain of *UGT2*. Black line represents intron. UDP-glycosyltransferase domain of *UGT2* gene in two species were completely identical. **b** *Cis*-acting element within 1000 bp upstream of the *UGT2* gene. Different colored rectangles represent different motifs. **c** Transient expression assay of luminescence intensity show the different transcription activity of *UGT2* in *F. tataricum* and *F. esculentum*. The color bar on the right represents the intensity of fluorescence. Representative images of *N. benthamiana* leaves 72 h after infiltration were shown

At the last step of the rutin synthesis pathway, however, *UGT2* exhibited differential expression between the two species (Fig. 3a). The *FtUGT2* expression level was obviously higher than *FeUGT2* across different tissues, especially in fruits (Fig. 3b). We further compared protein sequences of this gene based on the highly annotated haplotype genomes. We found that the UDP-glycosyltransferase domain regions in *UGT2* of two species are completely identical without amino acid mutation (Fig. 4a and Additional file 2: Fig. S16).

The enzyme activity in vitro of *FtUGT1*, *FeUGT1*, *FtUGT2*, and *FeUGT2* were performed for functional identification. Using quercetin and UDP-glucose (uridine diphosphate glucose) as substrates, the crude enzyme of gene *FtUGT1* and *FeUGT1* could produce isoquercitrin. Similarly, the enzyme of gene *FtUGT2* and *FeUGT2* could produce rutin using isoquercitrin and UDP-rhamnose (uridine diphosphate rhamnose) as substrates. This enzyme activity verification shows that the difference in rutin content between the two species is not due to the inactivation of key enzymes (Fig. 3d).

However, we found that the number, type, and distribution of *cis*-acting elements differed between the two species (Fig. 4b and Additional file 2: Fig. S17). We identified 30 *cis*-acting elements in the core promoter region of *F. tataricum* while only 24 for *F. esculentum* in the same homologous region (Supplemental Table 19 and Supplemental Table 20). The *cis*-acting elements of *F. esculentum* had the annotations “light responsive,” “zein metabolism regulation,” “MeJA-responsiveness,” “auxin response,” and “anoxic specific inducibility”. In *F. tataricum*, the annotations were “light response,” “salicylic acid responsiveness,” “anaerobic induction,” and “abscisic acid responsiveness” (Supplemental Table 19 and Supplemental Table 20). In addition, the average distance of the *cis-*acting elements of *F. esculentum* is closer to the transcription start site than in *F. tataricum* (Supplemental Table 19 and Supplemental Table 20). We used LUC imaging assays to verify the active ability of promoters in *FtUGT2* and *FeUGT2* gene in vivo. The result indicates that variations in the promoter region of *FtUGT2* gene confer it stronger active ability than that in *FeUGT2* (Fig. 4c). Thus, these changes in the *cis*-regulatory elements may have reduced the expression of *UGT2*, which finally led to the low rutin concentration.

### SRNase-based self-incompatibility of *F. esculentum*

**Fig. 5:**
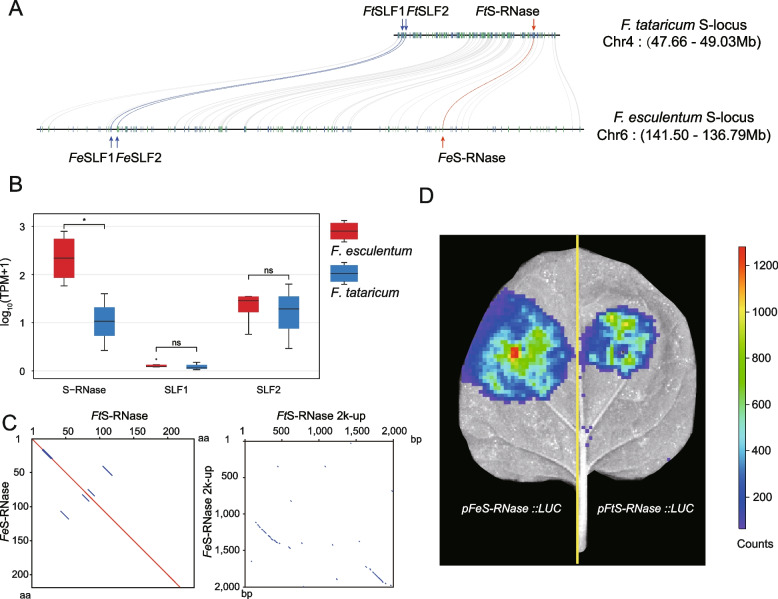
Genomic collinearity, expression and binding activities of self-incompatibility genes across the two species. **a** Genomic collinearity of three self-incompatibility genes. **b** Expressions of three self-incompatibility genes in flowers (significance was tested by paired *t* test *P-value < 0.05). **c** The differences in the regulatory regions between the two species. **d** Transient expression assay of luminescence intensity show the different transcription activity of *S-RNase* in *F. tataricum* and *F. esculentum*. The color bar on the right represents the intensity of fluorescence. Representative images of *N. benthamiana* leaves 72 h after infiltration were shown

Self-incompatibility is a widely occurring outcrossing mechanism to prevent inbreeding in plants; it provides a highly discriminatory pollen recognition and rejection system. *F. tataricum* is self-compatible whereas *F. esculentum* and other congeners are self-incompatible [19]. To search for *S-RNase* and S locus F-box (*SLF*) genes potentially involved in gametophytic self-incompatibility, the homologous genes in multiple species were used for homology searches. The *S-RNase*, *SLF1*, and *SLF2* genes were identified in the high-quality haplotype genomes of the two species. Three genes exhibited a good collinearity across the two species (Fig. 5a and Additional file 2: Fig. S18). Transcriptome profiling in flowers of the two species indicated that the *S-RNase* gene showed higher expression in *F. esculentum* than *F. tataricum*, while the expression of two *SLF* genes was not significantly different (Fig. 5b). Due to the highly similar S-RNase protein across the two species (Additional file 2: Fig. S19), we compared the upstream 2000 bps regulatory region sequences of the gene. We identified differences in the regulatory regions between the two species (Fig. 5c and Additional file 2: Fig. S20). We further used LUC imaging assays to verify the active ability of two contrasting promoters in vivo. In these assays, a plasmid (*pGreenII 0800-LUC*) containing the promoter of the *S-RNase* gene fused to the reporter gene luciferase was infiltrated into tobacco leaves. A strong luminescent signal was detected in the expression region of *pFeS-RNase::LUC*, but a much weaker luminescent signal was detected in *pFtS-RNase::LUC* (Fig. 5d). This demonstrated that the *F. esculentum S-RNase* promoter was stronger than the *F. tataricum S-RNase* promoter. The sequence mutations of the *S-RNase* promoter in *F. tataricum* caused in a significant decrease in gene expression, which may have resulted in the development of self-compatibility from self-incompatibility in this species.

## Discussion

We present here haplotype-resolved genomes at the chromosome level for two widely cultivated buckwheat crops. Compared to previously published draft genomes of these two species [1, 15], the genomes assembled in this study showed better continuity and integrity. The N50 of *F. tataricum* was significantly improved from 550.7 kb to 50 Mb, and the number of contigs was reduced from 8778 to 940. For *F. esculentum*, we overcame the difficulty in assembling the genome for this species, with its high heterozygosity and large size; the contig N50 was successfully improved from 25 k bp to 9.8 Mb. We demonstrated that LTR-RT insertions contributed greatly to the large genome size of *F. esculentum*. These haplotype genomes will serve as reliable references for subsequent breeding programs based on genetic variations. For example, combining genomic and phenotypic data will help identity putative genes involved in both environmental adaptation and crop production[20–22]. They also pave the way for studying the biology and evolution of the entire genus, especially between *F. esculentum* and *F. tataricum*.

We found that the promoter regions of two species at the gene *UGT2* showed great differences in expression profiles across different tissues, especially in the fruit. However, the other key genes in the rutin synthesis pathway show no distinct difference in expression. The coding regions of this gene are identical, without any distinct difference. However, the upstream regulatory regions of this gene show contrasting mutations that may have led to the high expression of the gene and high rutin concentration in *F. tataricum*. It remains unknown which is the ancient promoter region in the total genus without a comprehensive comparison with other species. Because high rutin concentration is only reported for *F. tataricum*, it is highly likely that domestication and breeding selected the allelic mutations of such a regulatory region that are further retained it in the current breeds of this species. Similarly, we also found that the mutations in the regulation region of the self-incompatibility *S-RNase* gene may have also resulted in selfing in *F. tataricum*. This gene is normally expressed in the outcrossing *F. esculentum* flowers, but is obviously reduced in the selfing *F. tataricum*. Our luciferase assay experiments confirmed that the noncoding changes in the promoter regulatory region reduced the activity of this gene in *F. tataricum* (Fig. 5). Most species of the genus *Fagopyrum* and other genera of the family Polygonaceae are outcrossing [9–11]. The selfing trait is obviously beneficial for crop cultivation because it does not need pollinators to set seeds [9–11]. Therefore, domestication and breeding may have led to the selection of selfing reproduction in *F. tataricum*.

Our high-quality genome sequences addressed well two contrasting cultivation traits between two closely related buckwheat crops. These results seem to suggest that *F. tataricum* may have experienced stronger artificial selection than *F. esculentum* in the history that leaded to the development of two beneficial traits through choosing the desired alleles in the non-coding promoter regulatory region. In fact, cultivated crops usually retain new advantageous traits because of the humans’ selection through allelic mutations in the promoter regulatory regions; this has been found for both rice and maize [20, 21, 23]. For example, the natural variations of the non-coding promoter of the gene to encode WD40 protein were artificially selected to increase the kernel row number in maize during domestication and artificial breeding [20, 21, 23]. In addition, in the wild *Arabidopsis thaliana* accessions, allelic variations in the cis-regulatory elements of one gene lead to its similar accumulation by different transcription factors under contrasted habitats. The accumulation of this gene further triggers different pathways to adapt the totally different environments through noncoding alleles [20, 21, 23]. These findings and our results together suggest that the non-coding alleles that usually determine expressions of the genes may have been frequently selected during artificial breeding the desired traits in many crops. Therefore, genetic manipulation of the non-coding promoter regions should be widely employed for breeding buckwheat and other crops in the future.

## Methods

### Plant material and tissue collection

Two buckwheat cultivars of *Fagopyrum esculentum* and *F. tataricum*, “Xinong9976” and “Qianku3”, were grown in a greenhouse of Sichuan University in Chengdu, Sichuan Province, China. Young leaves of “Xinong9976” and “Qianku3” were collected to extract high-quality DNA for Illumina and HiFi sequencing. For RNA extraction, six tissues including stems, leaves, roots, flowers, and young and mature seeds of the two species were collected and immediately frozen in liquid nitrogen. All sampling was repeated at least three times.

### DNA extraction, library construction, and genome sequencing

We sequenced the genomes with the PacBio HiFi Sequel II platform with two or one SMRTcells. High-molecular-weight DNA samples were isolated from 1.5 g of fresh leaves with a NucleoBond HMW DNA kit. Quality was assessed with a FEMTOpulse device and quantity measured by fluorometry Quantus. The HiFi libraries were prepared according to the manual “Procedure & Checklist—Preparing HiFi SMRTbell® Libraries using SMRTbell Express Template Prep Kit 2.0” with initial DNA fragmentation by g-Tubes (Covaris) and final library size binning by BluePippin. Size distribution was again controlled by FEMTOpulse (Agilent). Size-selected libraries were sequenced on a Sequel II device with Binding kit 2.0 and Sequel II Sequencing Kit 2.0 for 30 h. For Illumina sequencing, we generated ~ 50 × Illumina short reads on the HiSeq 2000 platform (Illumina, San Diego, CA). Raw sequencing data were trimmed to remove the adaptors and low-quality bases using Trimmomatic [24] after quality control by FastQC ( https://www.bioinformatics.babraham.ac.uk/projects/fastqc/).

Hi-C experiments were performed according to the manufacturer’s protocol. Briefly, 2 g of freshly harvested leaves was cut into 2–3 mm pieces and infiltrated in 2% formaldehyde before cross-linking was stopped by adding glycine. The tissue was ground to powder and suspended in nuclei isolation buffer to obtain a nuclei suspension. The procedure for the Hi-C experiment, including chromatin digestion, labeling of DNA ends, DNA ligation, purification, and fragmentation, was performed as described previously. The cross-linked DNA was digested with HindIII as previously described and marked by incubating with Klenow enzyme and biotin-14-dCTP overnight at 37 °C. The 5’ overhang of the fragments was repaired and labeled using biotinylated nucleotides, followed by ligation with T4 DNA polymerase. After reversal of cross-linking, ligated DNA was purified and sheared to 300–700 bp fragments using an S2 Focused-Ultrasonicator. The linked DNA fragments were enriched with streptavidin beads and prepared for Illumina HiSeq X Ten sequencing.

### Genome assembly and pseudo-chromosome scaffolding

The genome size of *F. tataricum* and *F. esculentum* was estimated using K-mer analysis. Briefly, K-mer counting was conducted using Jellyfish [25]. Genome size was estimated with GCE [26]. We assembled the haplotype genomes using high-quality long PacBio HiFi reads (15 × per haplotype) and hifiasm. For each haplotype genome, we mapped Hi-C data to the corresponding contigs using the Juicer v1.6.2 [27] pipeline and built primary scaffolds with 3D-DNA v180922 [28] with default parameters. Juicebox Assembly Tools v1.9.8 [29] was used to visualize and manually curate the assembly. We processed another round of scaffolding by 3D-DNA v180922 to obtain the final pseudo-chromosomes. Benchmarking Universal Single-Copy Orthologous gene analysis [30] (BUSCO) with the gene content of Embryophyta_odb10 was used to further evaluate the completeness of the assembled genome.

### Annotation of repeats

We combined homology alignment and de novo searches to identify repetitive sequences in genomes, as follows. We used RepeatMasker v 4.0.7. [31] to compare genomic sequences with published repetitive sequences in the Repbase v.16.10. RepeatModeler v1.0.10 [32] was used to construct a repeat library with the default parameters. RepeatMasker was then used again to search the whole genome for transposable elements with the repeat library trained by RepeatModeler. We integrated the results of the RepeatMasker, and removed those transposable elements with lower scores, and finally obtained the repeat annotation. Full-length long-terminal repeat retrotransposons (LTR-RTs) were initially identified using LTR_Finder v1.02 [33] and LTRharvest [34]. The LTR_retriever pipeline [35] was then used to integrate the results and remove false positive LTR-RTs. The time of insertion of LTR-RTs was estimated by LTR_retriever with the formula: *T* = *K*/2*r*, where *K* represents the genetic distance.

### Gene prediction and function annotation

Homology-based, transcriptome-based, and de novo approaches were used to predict high-quality protein-coding genes. For homology-based prediction, we used transcript protein sequences from previously published *Fagopyrum tataricum* (Pinku1) by GeneWise [36]. For ab initio annotation, Augustus [37] and GlimmerHMM [38] were employed. For transcriptome-based prediction, the transcriptome data were produced by Illumina sequencing of materials including leaves, stems, flowers, fruits, and roots. RNA-seq alignment files were generated using HISAT2 [39], and the PASA program [40] was used to align spliced transcripts and annotate candidate genes. Finally, we used EVidenceModeler [41] to combine gene models detected by these steps. After prediction, we used PASA again to update the gff3 file for three rounds to add alternatively spliced isoforms to gene models.

Functional annotation of protein-coding genes was achieved with BLASTp [42] (e-value 1e − 5 cutoff) using the SwissProt, TrEMBL [43], and NR databases. InterProScan [44] was used to annotate the protein domains by searching the InterPro database. GO terms for each gene were obtained from the corresponding annotation entries. Predicted proteins were carried by kofam-scan v1.3.0 [45] to obtain KO numbers for KEGG pathway annotation.

### Phylogenetic analyses

To investigate the evolutionary history of *Fagopyrum* species, seven other species with complete genomes, *A. vesiculosa*, *S. oleracea*, *B. vulgaris*, *H. ammodendron*, and *A. cruentus*, *H. undatus*, and *A. thaliana* were selected to use with OrthoFinder [46] using the default parameters to generate a matrix for phylogenetic analysis. Single-copy orthologs were identified from this dataset and used to construct a maximum likelihood phylogenetic tree. Protein sequences were aligned using MAFFT [47]. Gblocks [48] was used to extract conserved sites from multiple sequence alignment results. RAxML [49] was used to construct a phylogenetic tree taking *A. thaliana* as an outgroup; 1000 bootstrap analyses were performed to test the robustness of each branch. We further estimated the divergence times between species using the MCMCTree in the PAML [50] package. For the estimation of divergence time, we calibrated the model using the divergence time between *Fagopyrum* and *A. thaliana* (112.4–125.0 Mya) obtained from the TimeTree database (http://www.timetree.org/).

Gene families that underwent expansion or contraction were identified in the nine sequenced species using CAFÉ [51] (Computational Analysis of gene Family Evolution). Homologous pairs of nine species’ proteins were identified using an all-to-all search in BLASTp with an e-value cutoff of 1e − 5. WGDI [52] with “-icl” parameters used to identify collinear blocks, each containing at least seven collinear gene pairs. To look for polyploidy events, the “-ks” parameter of WGDI was used to calculate *Ks* using the PAML package between collinear genes in each pair from: within *F. esculentum* and *F. tataricum*; between *F. esculentum* and *F. tataricum*; between *F. esculentum* and *A. vesiculosa*; and between *F. tataricum* and *A. vesiculosa.*

### Predication and annotation of rutin biosynthesis genes

The published Rutin bio-synthesis related gene sequences of *Fagopyrum tataricum* were retrieved from NCBI and used as queries in BLASTp searches against the haplotype genome assemblies of the two species. Candidate hit pairs with at least 90% coverage and 90% identity were treated as homologous genes. All homologous genes were further confirmed by hmmsearch against the Pfam database.

### Functional identification of UGT genes in vitro

The coding sequence of *FeUGT1*, *FeUGT2*, *FtUGT1*, and *FtUGT2* were cloned and inserted into the PET30b( +) expression vector, and then recombinant plasmid of these four genes were transformed into *E. coli* Rosetta (DE3) (Tsingke Biotechnology Co., Ltd., Beijing). pET30b( +)-transformed *E. coli* Rosetta (DE3) cells were treated in parallel as a control. Four recombinant proteins were extracted by ultrasonic cell breaker (on/off: 3 s/7 s, power: 90%), purified using nickel-nitrilotriacetic acid (Ni–NTA) agarose, and eluted with 250 mM imidazole. After concentrating, each 5 ug of the four purified proteins were incubated at 30 °C with 100 mM Tris–HCl (pH 8.0), 100 mM Tris–HCl (pH 7.5), 14 mM β-mercatoethanol, 4 mM UDP-rhamnose or UDP-glucose, and 0.1 mM substrate for 30 min and reaction was stopped by adding methanol. Glycosylated products were detected using a LC–ESI–MS/MS system (LC, Shimadzu LC30AD; MS, QTRAP 6500 +) with a Thermo Hypersil Gold analytical column (100 × 2.1 mm, 1.9 μm). Data analysis was performed using Analyst 1.7.0. Standards of quercetin, isoquercitrin, and rutin were purchased from Yuanye Bio-Technology (Shanghai, China).

### Identification and comparison of self-incompatibility genes

The sequences of the S-locus haplotypes were identified from the *Fagopyrum tataricum* and *Fagopyrum esculentum* assembled genome using the homologous genes of *S-RNase* in other species downloaded from NCBI. Best matches were identified using BLASTp with an e-value cutoff of 1e − 5. InterProScan was then used to characterize genes containing an F-box domain and an F-box-associated motif based on data acquired from Pfam, SMART, PANTHER, and PRINTS. The syntenic regions of the S-locus in *Fagopyrum tataricum* and *Fagopyrum esculentum* were identified by JCVI [53].

### Gene expression and normalization

Clean reads of six tissues of two species were aligned to haplotype genomes by HISAT2 software. The TPM (transcripts per million) was calculated by StringTie [54] software. Protein-coding genes with one-to-one orthologs in four haplotypes of two species were identified to compare the expression profiles. All normalized expression values of the one-to-one orthologous genes were then TMM normalized by the edgeR [55] package between all tissues in all haplotypes. All TPM values between all samples of four haplotypes were normalized using the calculated normalized expression values. And edgeR was used to detect differentially expressed genes (DEGs) in two *Fagopyrum* species.

### Dual-luciferase assay

For the dual-luciferase assay, the promoter constructions inserted into *pGreenII 0800-LUC* were used in the analysis. The *A. tumefaciens* GV3101 strains harboring the promoter were cultured at 28 °C overnight. The resuspension buffer (10 mM MgCl_2_, 10 mM MES, and 100 mM acetosyringone) was used to dilute the cultures to an OD600 of 0.6. The *pFeUGT2::LUC, pFtUGT2::LUC*, *pFeS-RNase::LUC*, and *pFtS-Rnase::LUC* were injected into separate *N. benthamiana* leaves and then cultured 2 days in the dark and 1 day in the light at 25 °C. The injected leaves were then detached and sprayed with 1 mM D-Luciferin sodium salt (Solarbio Beijing) + 0.01%Triton X-100. The luciferase luminescence from the infiltrated area was imaged using the IVIS Lumina III In Vivo Imaging System (PerKinElmer, Germany).

## Supplementary Information

**Additional file 1: Fig. S1.** PacBio long reads (2 cell) length distribution of *F. esculentum*. **Fig. S2.** Genome size and heterozygosity estimation for *F. esculentum*. **Fig. S3.** Hi-C map of the Fe-haplotype 1 showing genome-wide all-by-all interactions. The map shows a high resolution of individual chromosomes that are scaffolded and assembled independently. **Fig. S4.** Hi-C map of the Fe-haplotype 2 showing genome-wide all-by-all interactions. The map shows a high resolution of individual chromosomes that are scaffolded and assembled independently. **Fig. S5.** Genome size and heterozygosity estimation for *F. tataricum*. **Fig. S6.** PacBio long reads (1 cell) length distribution of *F. tataricum*. **Fig. S7.** Hi-C map of the Ft-haplotype 1 showing genome-wide all-by-all interactions. The map shows a high resolution of individual chromosomes that are scaffolded and assembled independently. **Fig. S8.** Hi-C map of the Ft-haplotype 2 showing genome-wide all-by-all interactions. The map shows a high resolution of individual chromosomes that are scaffolded and assembled independently. **Fig. S9.** Genome alignment between *F. tataricum* cv. Pinku1 and Ft-haplotype 1. **Fig. S10.** Genome alignment between *F. tataricum* cv. Pinku1 and Ft-haplotype 2.

**Additional file 2: Fig. S11.** Syntenic block dotplot within *F. esculentum* genome. **Fig. S12.** Syntenic block dotplot between *F. esculentum* and *S. oleracea* genomes. **Fig. S13.** Gene ontology (GO) enrichment analysis of the expanded gene families in *F. tataricum*. **Fig. S14.** GO enrichment analysis of the expanded gene families in *F. esculentum*. **Fig. S15.** Overview of the rutin biosynthetic pathway in *F. tataricum* and *F. esculentum* with expression profiles of key enzyme genes. **Fig. S16.** Multiple sequence alignment of the UGT2 proteins for the 4 assemblies. Red box indicates the position of UDP-glycosyltransferase functional domian (PF00201). **Fig. S17.** Sequence alignment of UGT2 promoter sequences in *F. tataricum* and *F. esculentum* haplotyped genomes. **Fig. S18.** Gene collinear relationship between *F. tataricum* (n=8) and *F. esculentum* (n=8) genomes. Red lines indicate S-RNase genes loci while blue lines indicate SLF genes loci. **Fig. S19** Multiple sequence alignment of the S-RNase proteins for the 2 assemblies. **Fig. S20** Sequence alignment of S-RNase promoter sequences in *F. tataricum* and *F. esculentum* genomes. **Fig. S21.** The number of different families within the *Copia* (a) and *Gypsy* (b) superfamilies. **Fig. S22.** The genome comparison between the 2 Mb to 3 Mb interval of Chromosome 8 of Fe-haplotype 1 and FES_r1.0.

**Additional file 3: Table S1.** Sequencing reads used for assembly of *F. esculentum* genome. **Table S2.** Sequencing reads used for assembly of *F. tataricum* genome. **Tables S3.** BUSCO analysis of genome assembly completeness of *F. esculentum* and *F. tataricum*. **Table S4.** Classification of repetitive elements in *F. esculentum* and *F. tataricum* genomes. **Table S5.** Summary of RNA sequencing data. **Table S6.** Gene model characteristics of *F. esculentum* genome. **Tables S7.** BUSCO evaluation of predicted gene models for two *Fagopyrum* genomes. **Table S8.** Gene model characteristics of *F. tataricum* genome. **Table S9.** Functional annotation of predicted gene for *F. esculentum* genome. **Table S10.** Functional annotation of predicted gene for *F. tataricum* genome. **Table S11.** Gene data sets used for comparative genomic analysis. **Table S12.** Syntenic gene pairs within *F. esculentum* genome. **Table S13.** Syntenic gene pairs within *F. tataricum* genome. **Table S14.** Summary of gene family clustering. **Table S15.** GO enrichment analysis of lineage-specific genes in the *Fagopyrum*. **Table S16.** Summary of gene families expansion/contraction in species. **Table S17.** GO enrichment analysis of the expanded gene families in *F. tataricum*. **Table S18.** GO enrichment analysis of the expanded gene families in *F. esculentum*. **Table S19.** Summary of *FeUGT2* promoter cis-acting elements prediction. **Table S20.** Summary of *FtUGT2* promoter cis-acting elements prediction. **Table S21.** The list of S-RNase in different species. **Table S22.** The genetic differences within the haploid genome.

## Data Availability

The PacBio long reads and short reads of *Fagopyrum esculentum* and *Fagopyrum tataricum* were uploaded to the NCBI BioProject database under accession numbers PRJNA937607 [56] and PRJNA935840 [57], respectively. The final chromosome-scale genome assembly were available in Figshare [58].
