# Supplementary material for: Haplotype-resolved genomes of two buckwheat crops provide insights into their contrasted rutin concentrations and reproductive systems
Source: BMC Biol. 2023 Apr 17;21:87. doi: 10.1186/s12915-023-01587-1 (PMC10111841; doi:10.1186/s12915-023-01587-1)
Supplement: Supplementary file 1 — Additional file 1: Fig. S1. PacBio long reads (2 cell) length distribution of F. esculentum. Fig. S2. Genome size and heterozygosity estimation for F. esculentum. Fig. S3. Hi-C map of the Fe-haplotype 1 showing genome-wide all-by-all interactions. The map shows a high resolution of individual chromosomes that are scaffolded and assembled independently. Fig. S4. Hi-C map of the Fe-haplotype 2 showing genome-wide all-by-all interactions. The map shows a high resolution of individual chromosomes that are scaffolded and assembled independently. Fig. S5. Genome size and heterozygosity estimation for F. tataricum. Fig. S6. PacBio long reads (1 cell) length distribution of F. tataricum. Fig. S7. Hi-C map of the Ft-haplotype 1 showing genome-wide all-by-all interactions. The map shows a high resolution of individual chromosomes that are scaffolded and assembled independently. Fig. S8. Hi-C map of the Ft-haplotype 2 showing genome-wide all-by-all interactions. The map shows a high resolution of individual chromosomes that are scaffolded and assembled independently. Fig. S9. Genome alignment between F. tataricum cv. Pinku1 and Ft-haplotype 1. Fig. S10. Genome alignment between F. tataricum cv. Pinku1 and Ft-haplotype 2. [file 12915_2023_1587_MOESM1_ESM.docx]

**Haplotype-resolved genomes of two buckwheat crops provide insights into their contrasted rutin concentrations and reproductive systems**

Hao Lin^1,2†^, Yingjun Yao^1†^, Pengchuan Sun^1^, Landi Feng^1^, Shuo Wang^1^, Yumeng Ren^1^, Xi Yu^1^, Zhengxiang Xi^1^, Jianquan Liu^1,3*^

^1^Key Laboratory for Bio-Resource and Eco-Environment of Ministry of Education & Sichuan Zoige Alpine Wetland Ecosystem National Observation and Research Station, College of Life Science, Sichuan University, Chengdu, China;

^2^State Key Laboratory of Dao-di Herbs, Beijng, 100700, P. R. China;

^3^State Key Laboratory of Herbage Improvement and Grassland Agro‐Ecosystems, College of Ecology, Lanzhou University, Lanzhou 730000, China

†Hao Lin and Yingjun Yao contributed equally to this work.

*Correspondence: Jianquan Liu ([liujq@nwipb.ac.cn](mailto:liujq@nwipb.ac.cn))


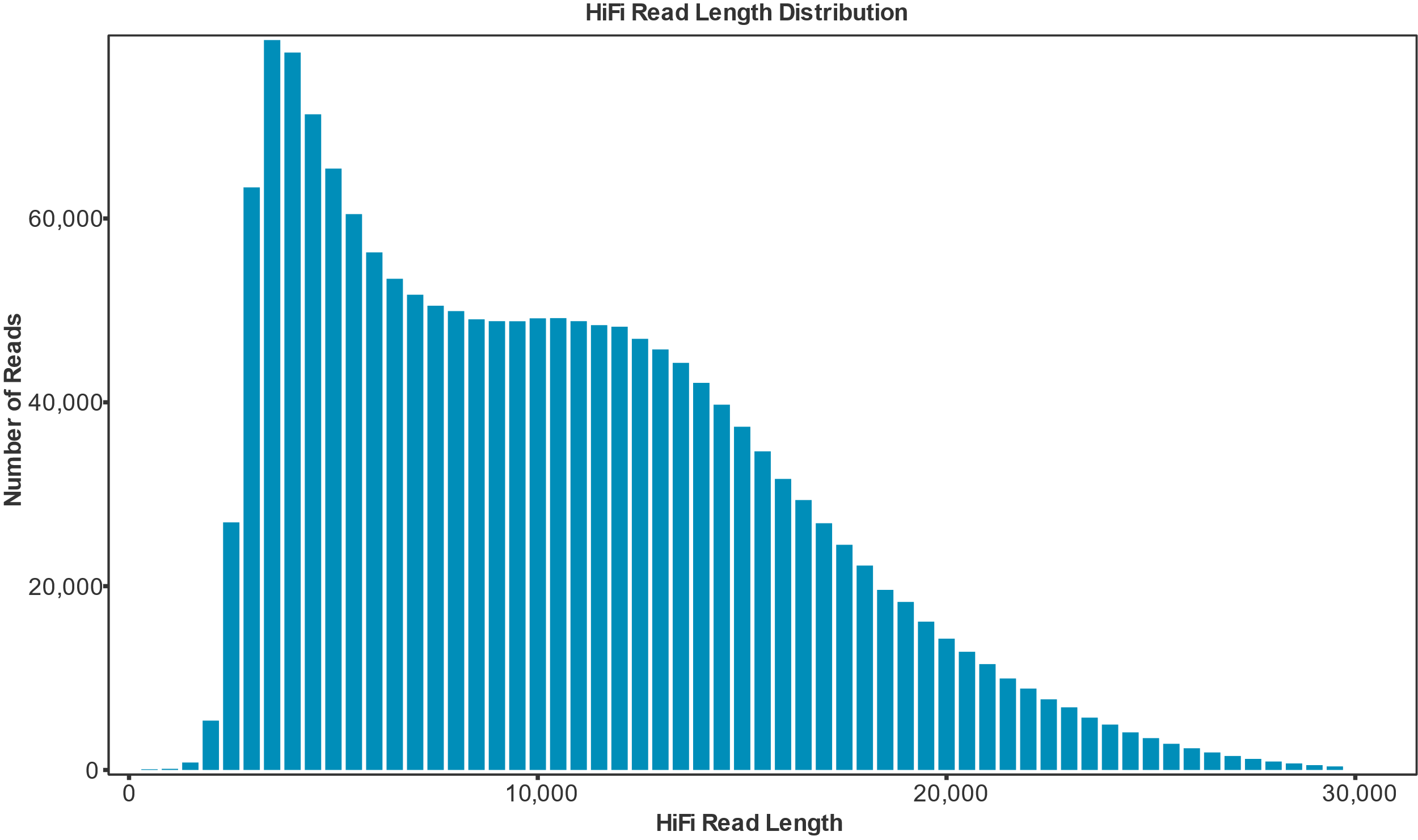


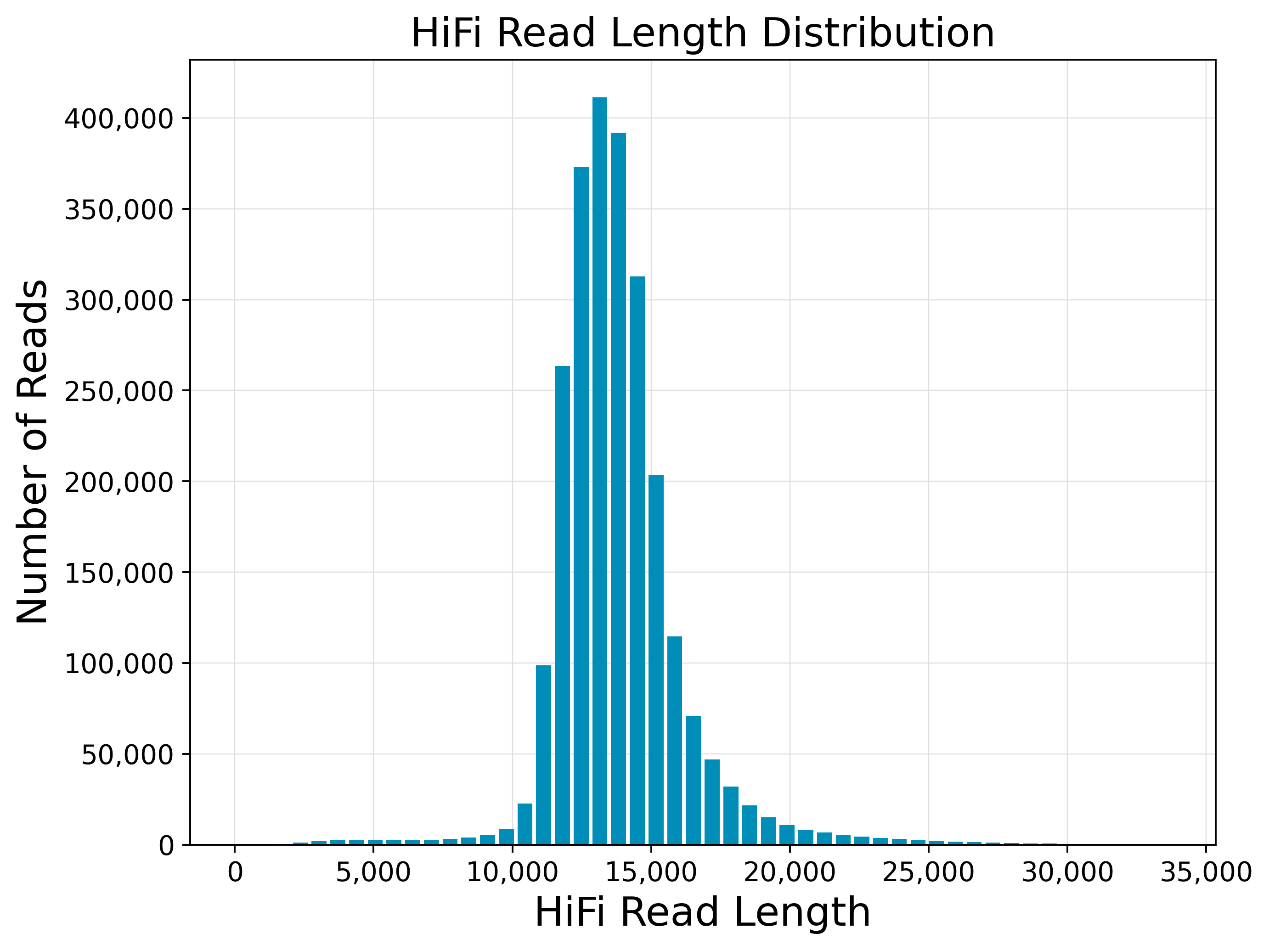


**Fig. S1** PacBio long reads (2 cell) length distribution of *F. esculentum*.


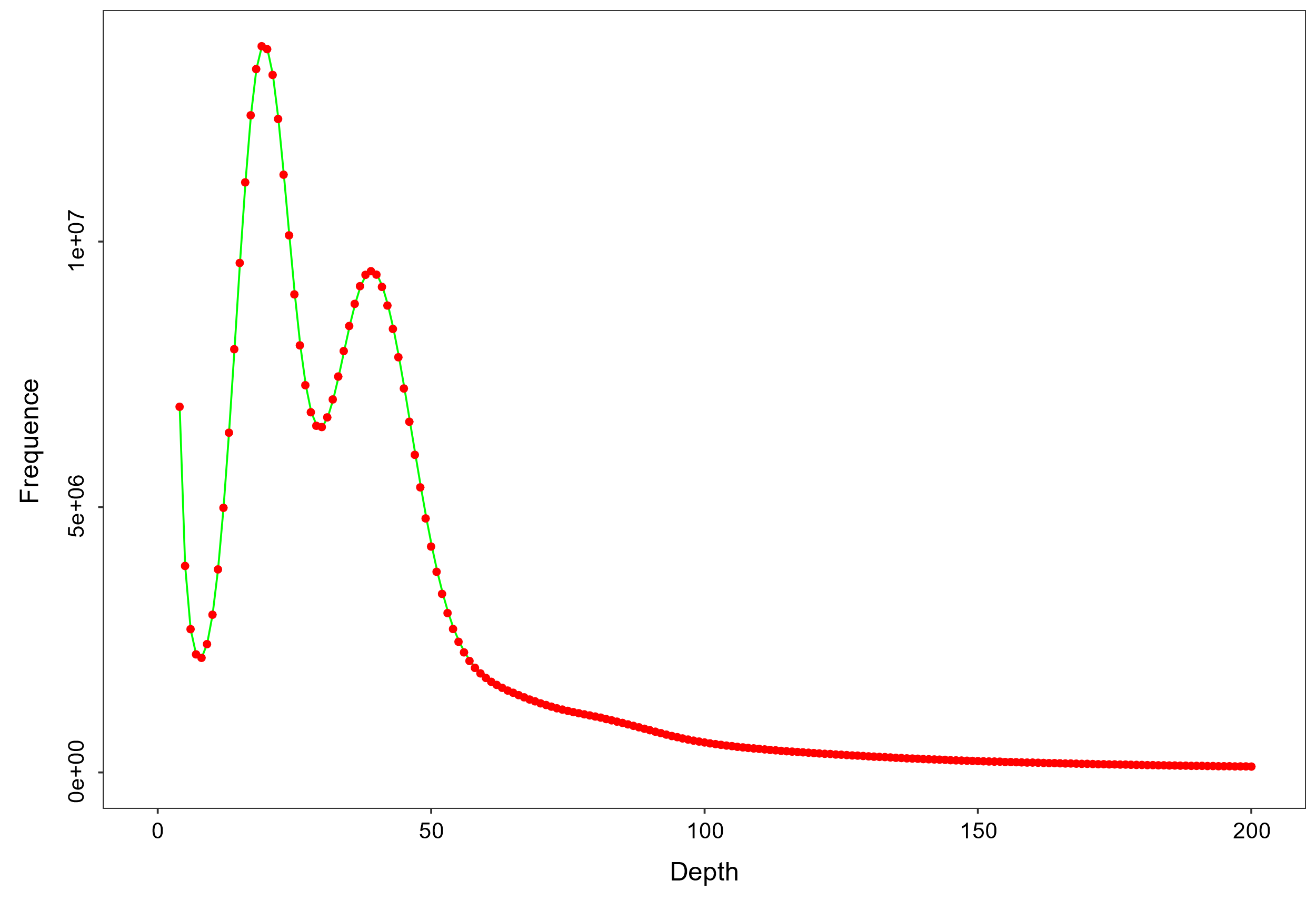


**Fig. S2.** Genome size and heterozygosity estimation for *F. esculentum*.


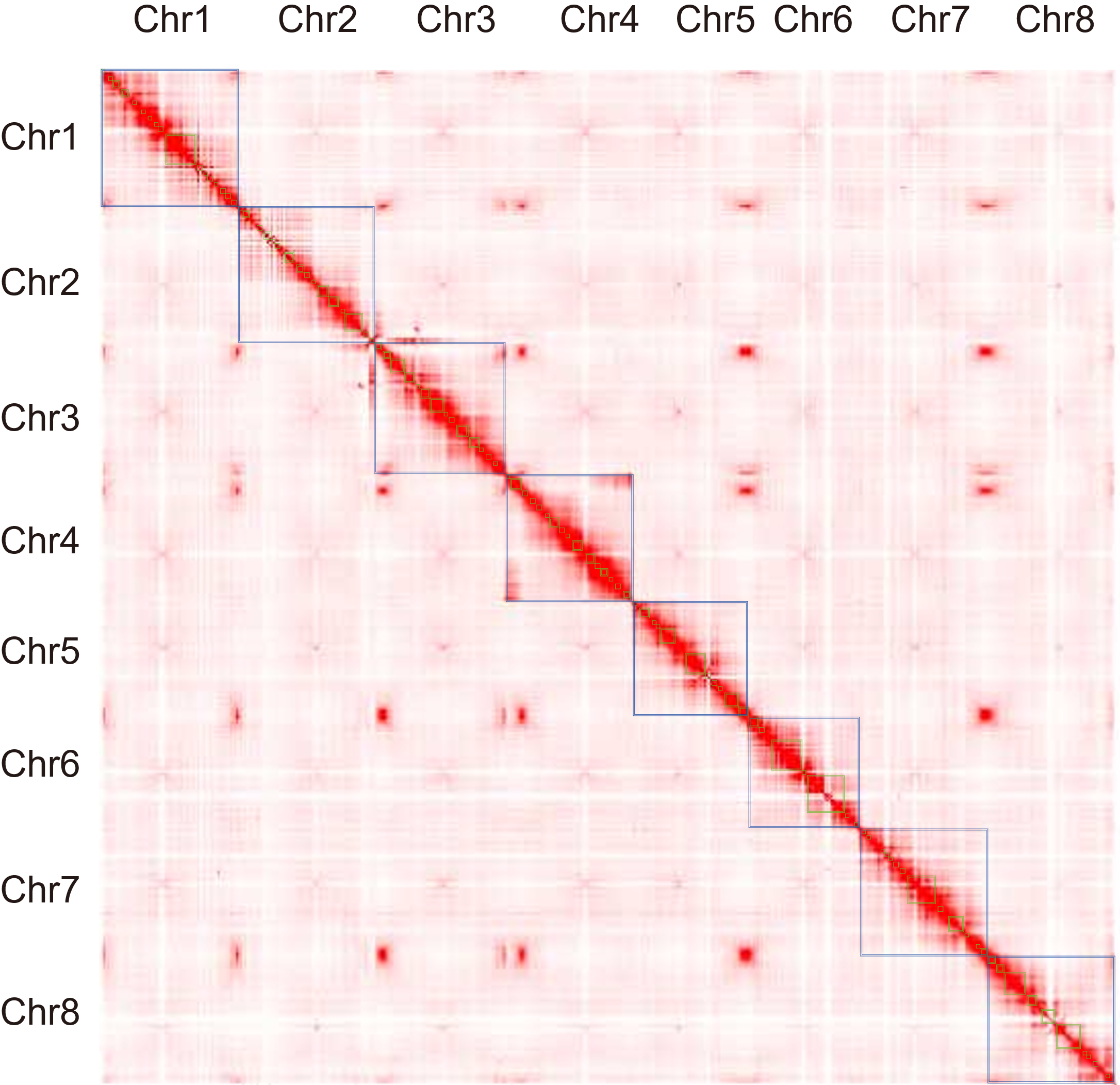


**Fig. S3.** Hi-C map of the Fe-haplotype 1 showing genome-wide all-by-all interactions. The map shows a high resolution of individual chromosomes that are scaffolded and assembled independently.


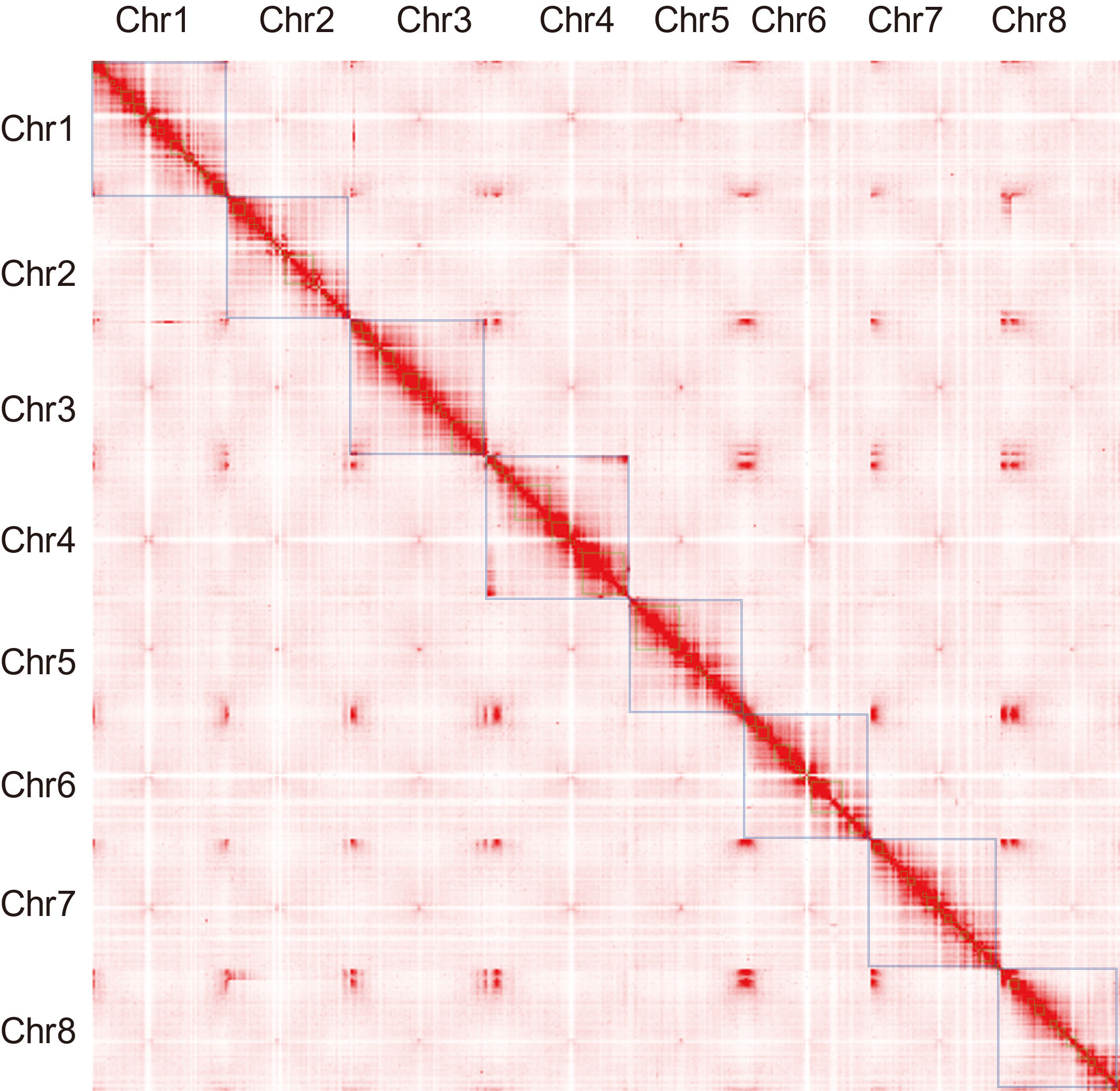


**Fig. S4.** Hi-C map of the Fe-haplotype 2 showing genome-wide all-by-all interactions. The map shows a high resolution of individual chromosomes that are scaffolded and assembled independently.


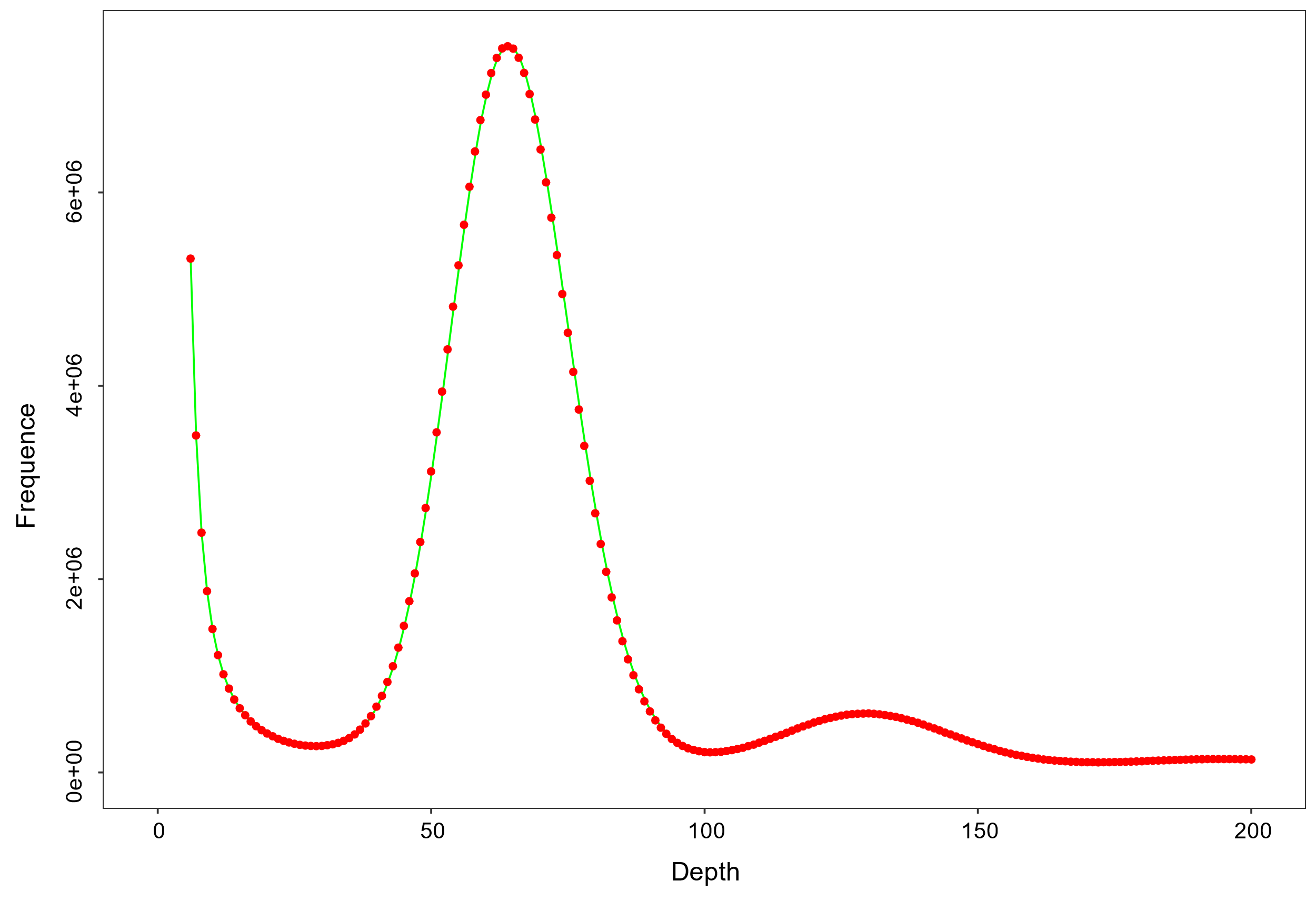


**Fig. S5.** Genome size and heterozygosity estimation for *F. tataricum*.


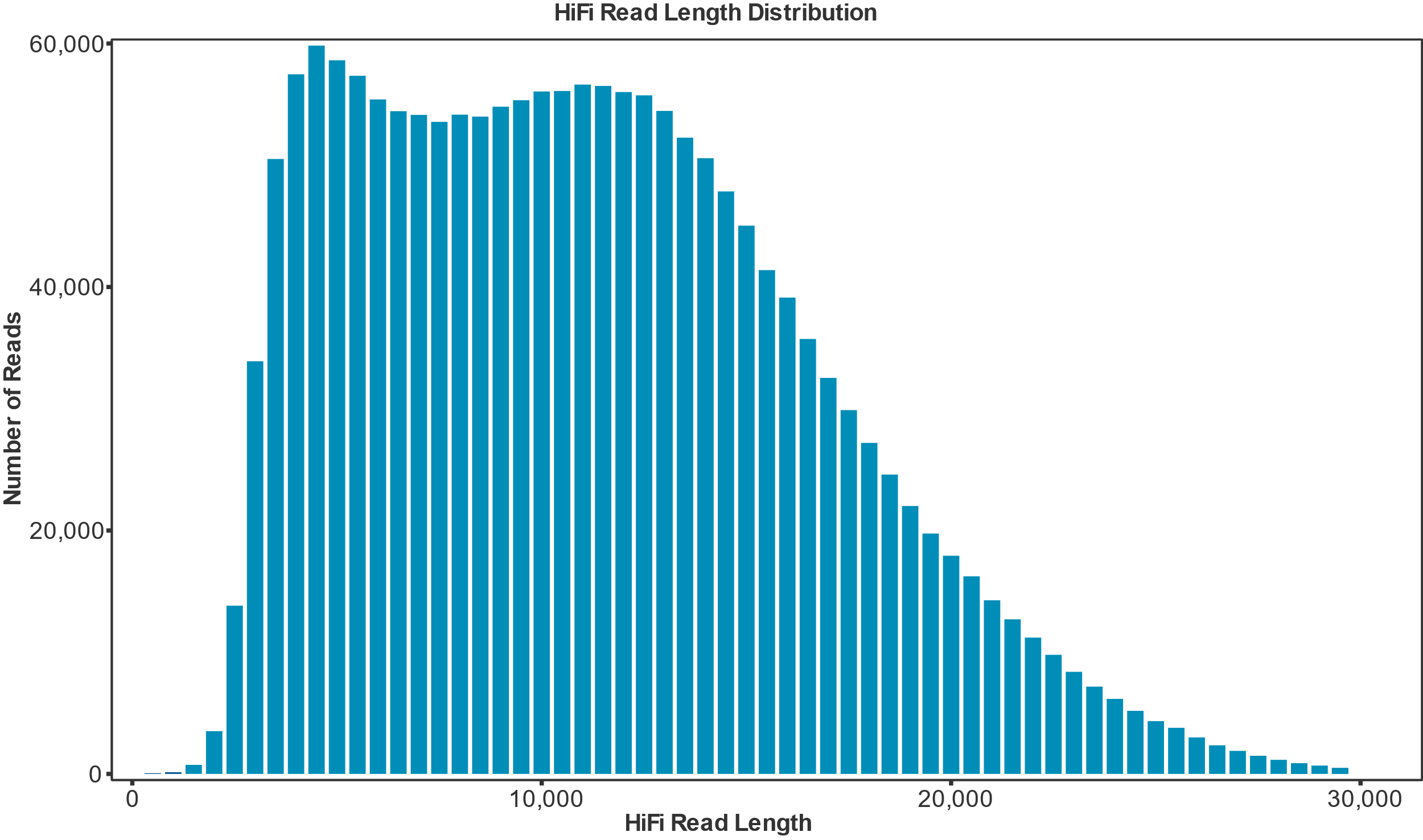


**Fig. S6** PacBio long reads (1 cell) length distribution of *F. tataricum*.


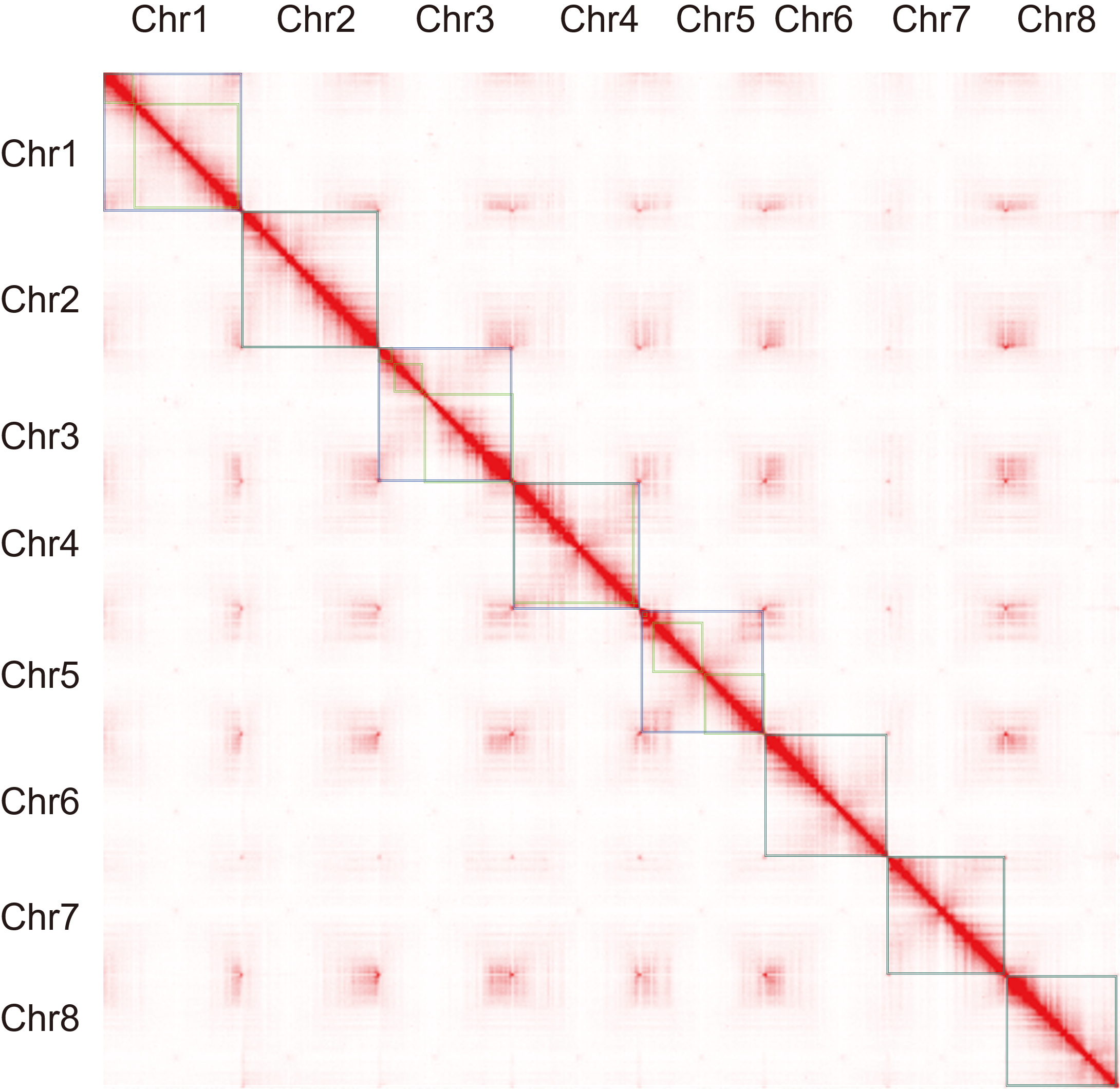


**Fig. S7** Hi-C map of the Ft-haplotype 1 showing genome-wide all-by-all interactions. The map shows a high resolution of individual chromosomes that are scaffolded and assembled independently.


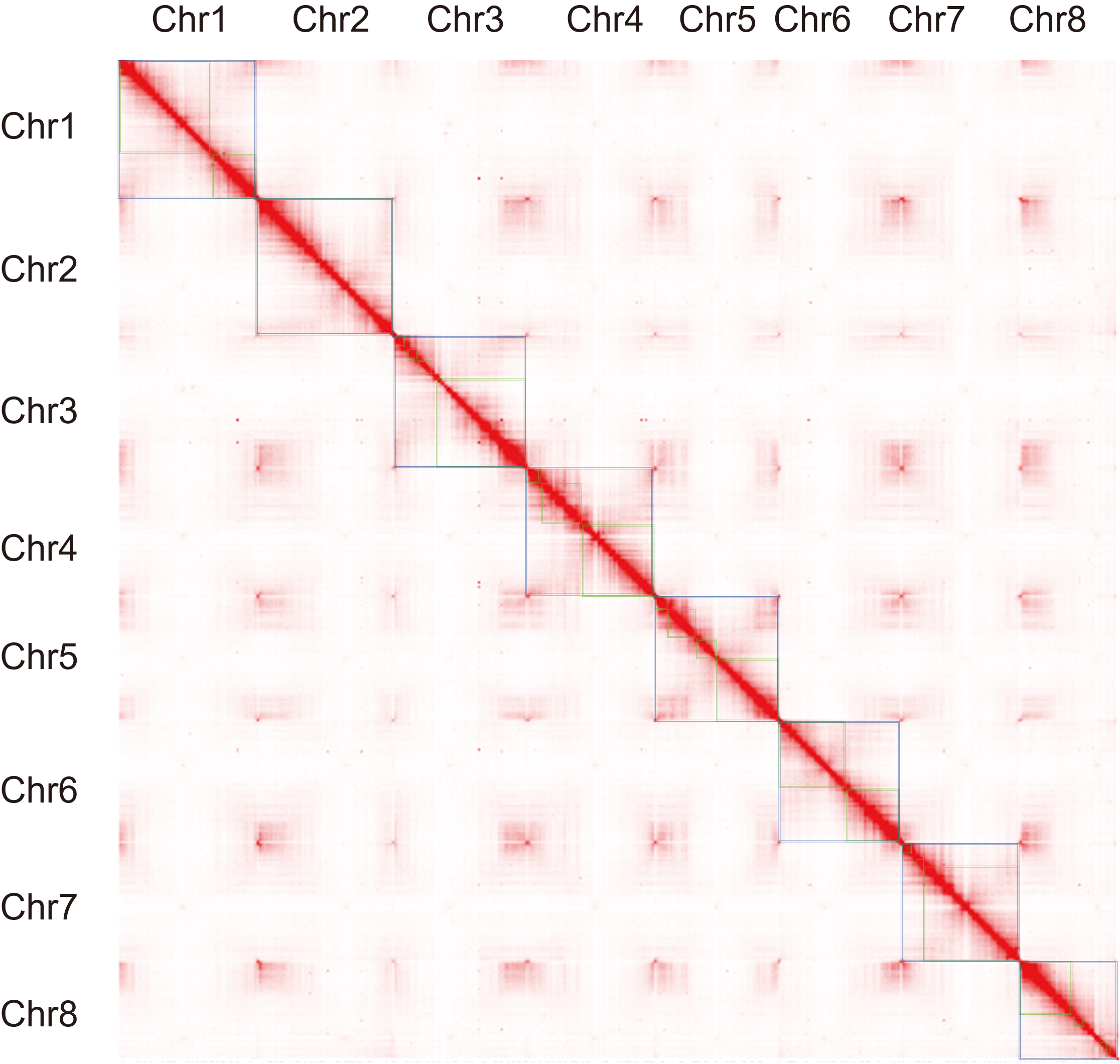


**Fig. S8** Hi-C map of the Ft-haplotype 2 showing genome-wide all-by-all interactions. The map shows a high resolution of individual chromosomes that are scaffolded and assembled independently.


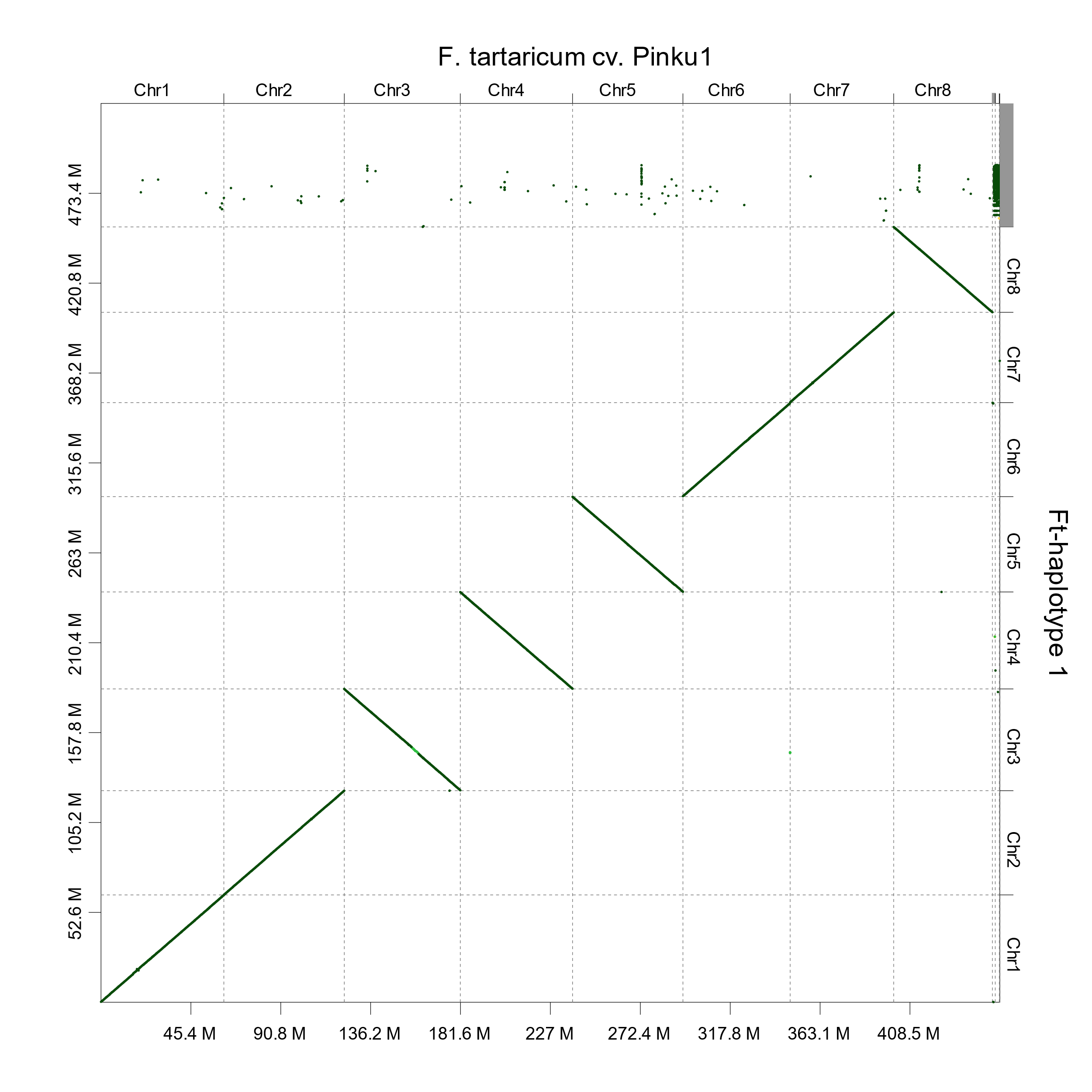


**Fig. S9** Genome alignment between *F. tataricum* cv. Pinku1 and Ft-haplotype 1.


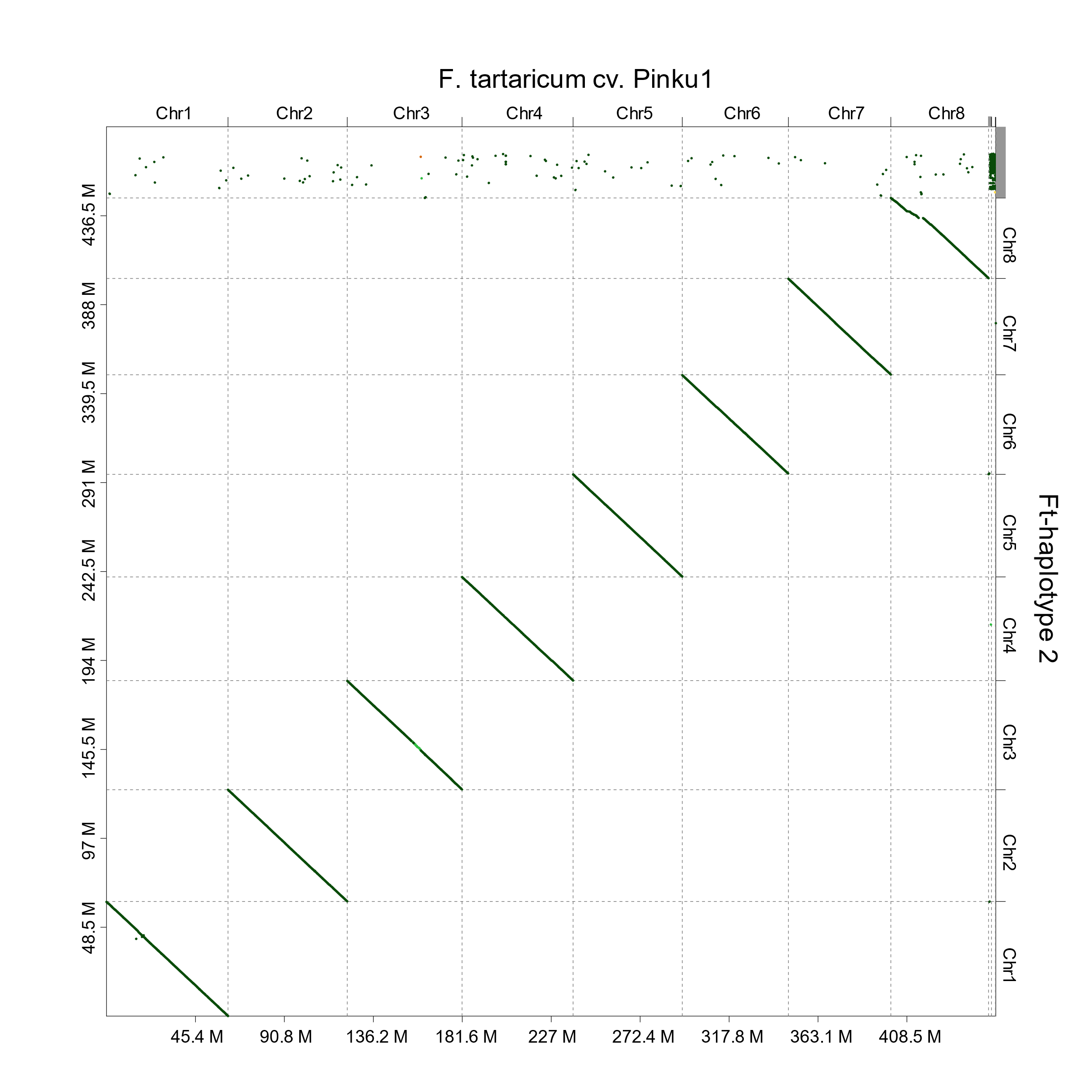


**Fig. S10** Genome alignment between *F. tataricum* cv. Pinku1 and Ft-haplotype 2.
