# Supplementary material for: Haplotype-resolved genomes of two buckwheat crops provide insights into their contrasted rutin concentrations and reproductive systems
Source: BMC Biol. 2023 Apr 17;21:87. doi: 10.1186/s12915-023-01587-1 (PMC10111841; doi:10.1186/s12915-023-01587-1)
Supplement: Supplementary file 2 — Additional file 2: Fig. S11. Syntenic block dotplot within F. esculentum genome. Fig. S12. Syntenic block dotplot between F. esculentum and S. oleracea genomes. Fig. S13. Gene ontology (GO) enrichment analysis of the expanded gene families in F. tataricum. Fig. S14. GO enrichment analysis of the expanded gene families in F. esculentum. Fig. S15. Overview of the rutin biosynthetic pathway in F. tataricum and F. esculentum with expression profiles of key enzyme genes. Fig. S16. Multiple sequence alignment of the UGT2 proteins for the 4 assemblies. Red box indicates the position of UDP-glycosyltransferase functional domian (PF00201). Fig. S17. Sequence alignment of UGT2 promoter sequences in F. tataricum and F. esculentum haplotyped genomes. Fig. S18. Gene collinear relationship between F. tataricum (n=8) and F. esculentum (n=8) genomes. Red lines indicate S-RNase genes loci while blue lines indicate SLF genes loci. Fig. S19 Multiple sequence alignment of the S-RNase proteins for the 2 assemblies. Fig. S20 Sequence alignment of S-RNase promoter sequences in F. tataricum and F. esculentum genomes. Fig. S21. The number of different families within the Copia (a) and Gypsy (b) superfamilies. Fig. S22. The genome comparison between the 2 Mb to 3 Mb interval of Chromosome 8 of Fe-haplotype 1 and FES_r1.0. [file 12915_2023_1587_MOESM2_ESM.docx]

**Haplotype-resolved genomes of two buckwheat crops provide insights into their contrasted rutin concentrations and reproductive systems**

Hao Lin^1,2†^, Yingjun Yao^1†^, Pengchuan Sun^1^, Landi Feng^1^, Shuo Wang^1^, Yumeng Ren^1^, Xi Yu^1^, Zhengxiang Xi^1^, Jianquan Liu^1,3*^

^1^Key Laboratory for Bio-Resource and Eco-Environment of Ministry of Education & Sichuan Zoige Alpine Wetland Ecosystem National Observation and Research Station, College of Life Science, Sichuan University, Chengdu, China;

^2^State Key Laboratory of Dao-di Herbs, Beijng, 100700, P. R. China;

^3^State Key Laboratory of Herbage Improvement and Grassland Agro‐Ecosystems, College of Ecology, Lanzhou University, Lanzhou 730000, China

†Hao Lin and Yingjun Yao contributed equally to this work.

*Correspondence: Jianquan Liu ([liujq@nwipb.ac.cn](mailto:liujq@nwipb.ac.cn))


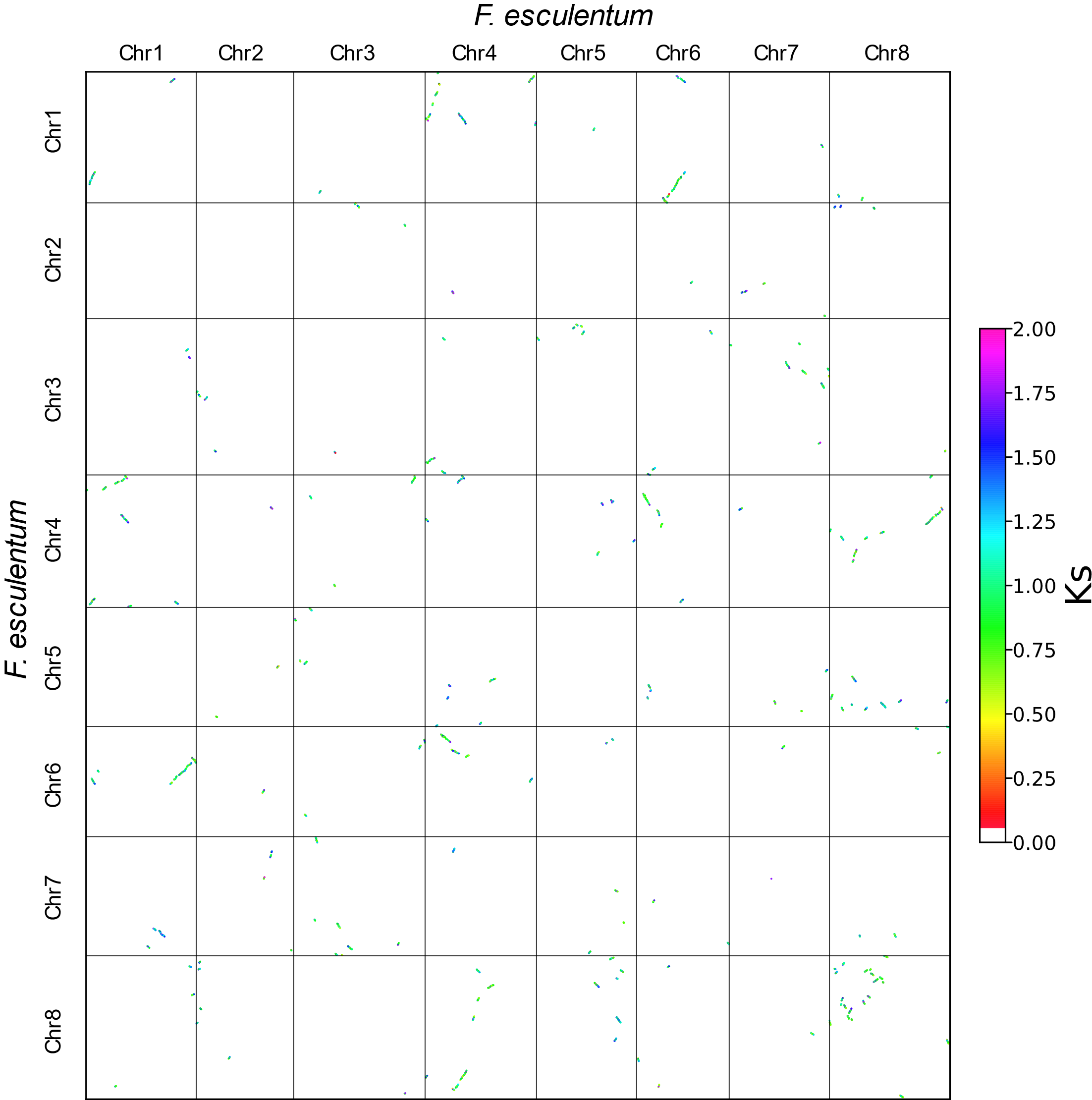


**Fig. S11** Syntenic block dotplot within *F. esculentum* genome.


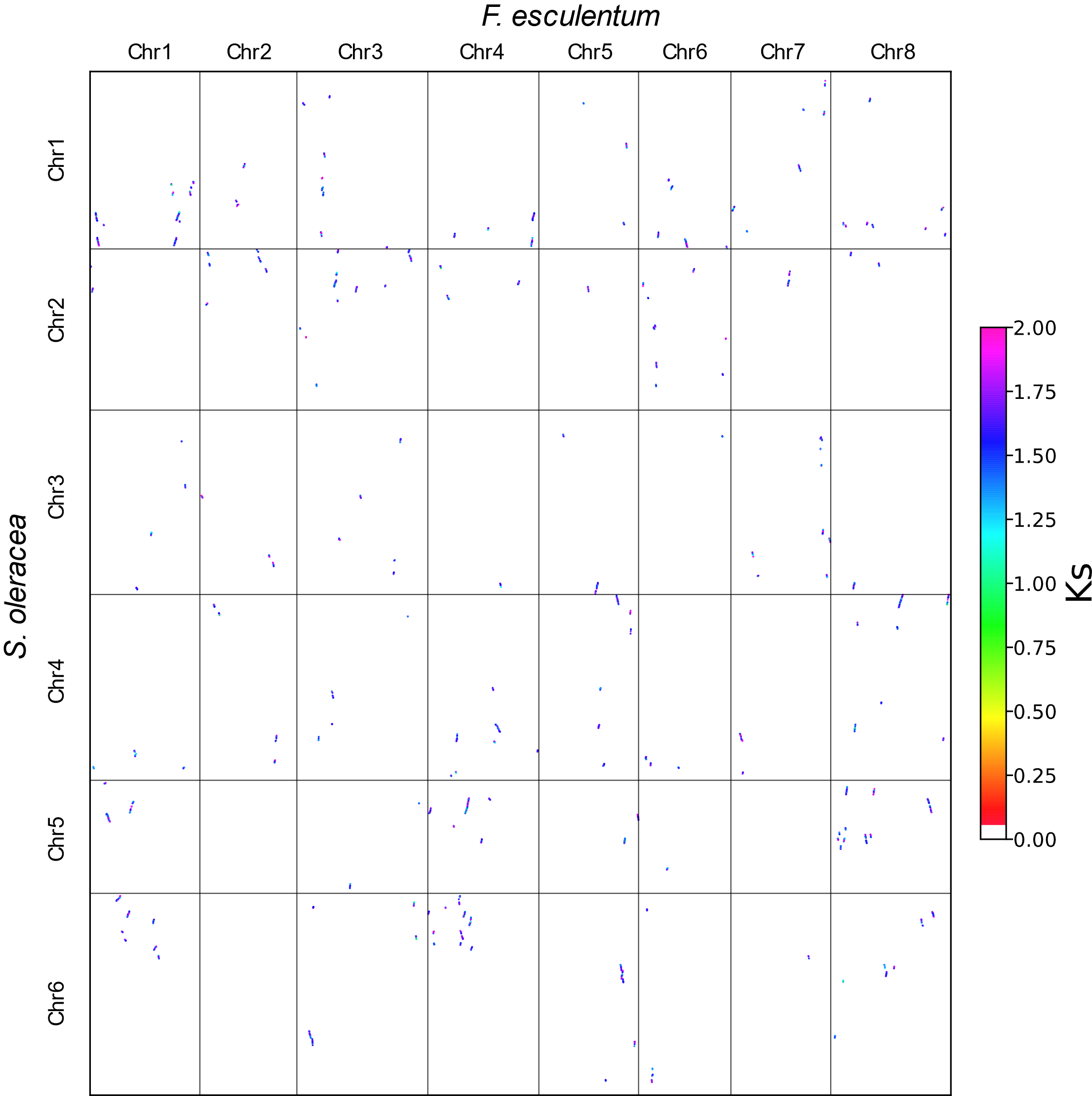


**Fig. S12** Syntenic block dotplot between *F. esculentum* and *S. oleracea* genomes.


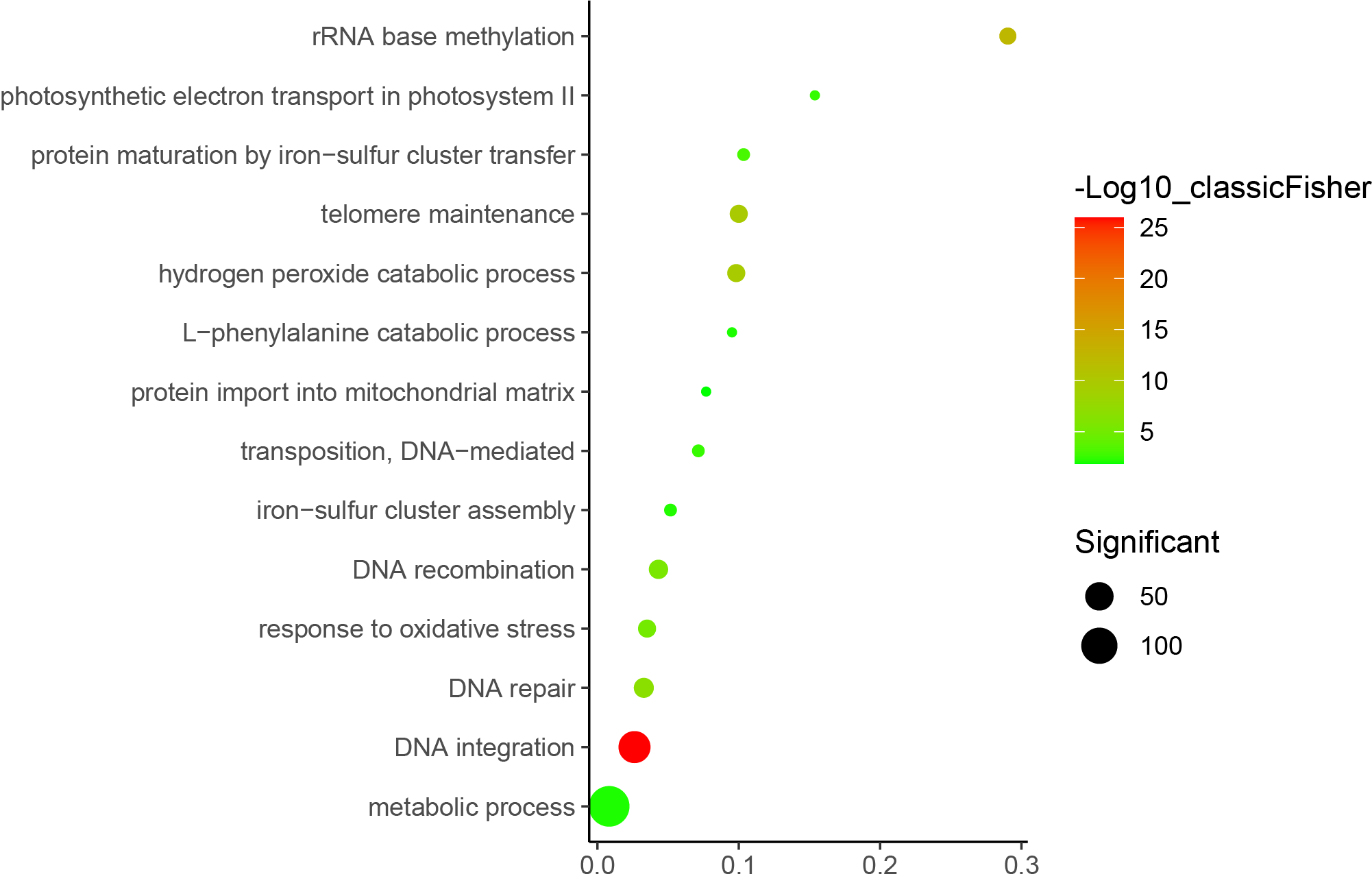


**Fig. S13.** Gene ontology (GO) enrichment analysis of the expanded gene families in *F. tataricum*.


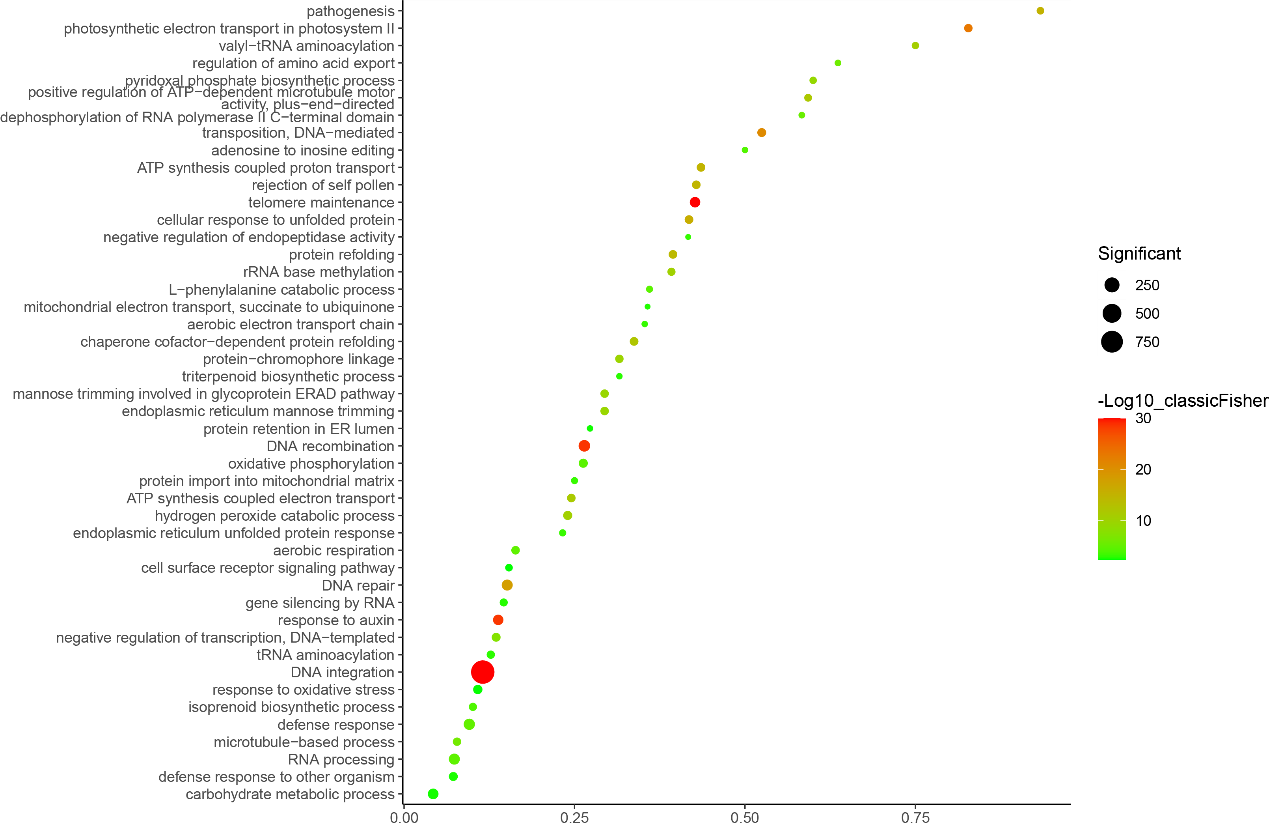


**Fig. S14** GO enrichment analysis of the expanded gene families in *F. esculentum*.


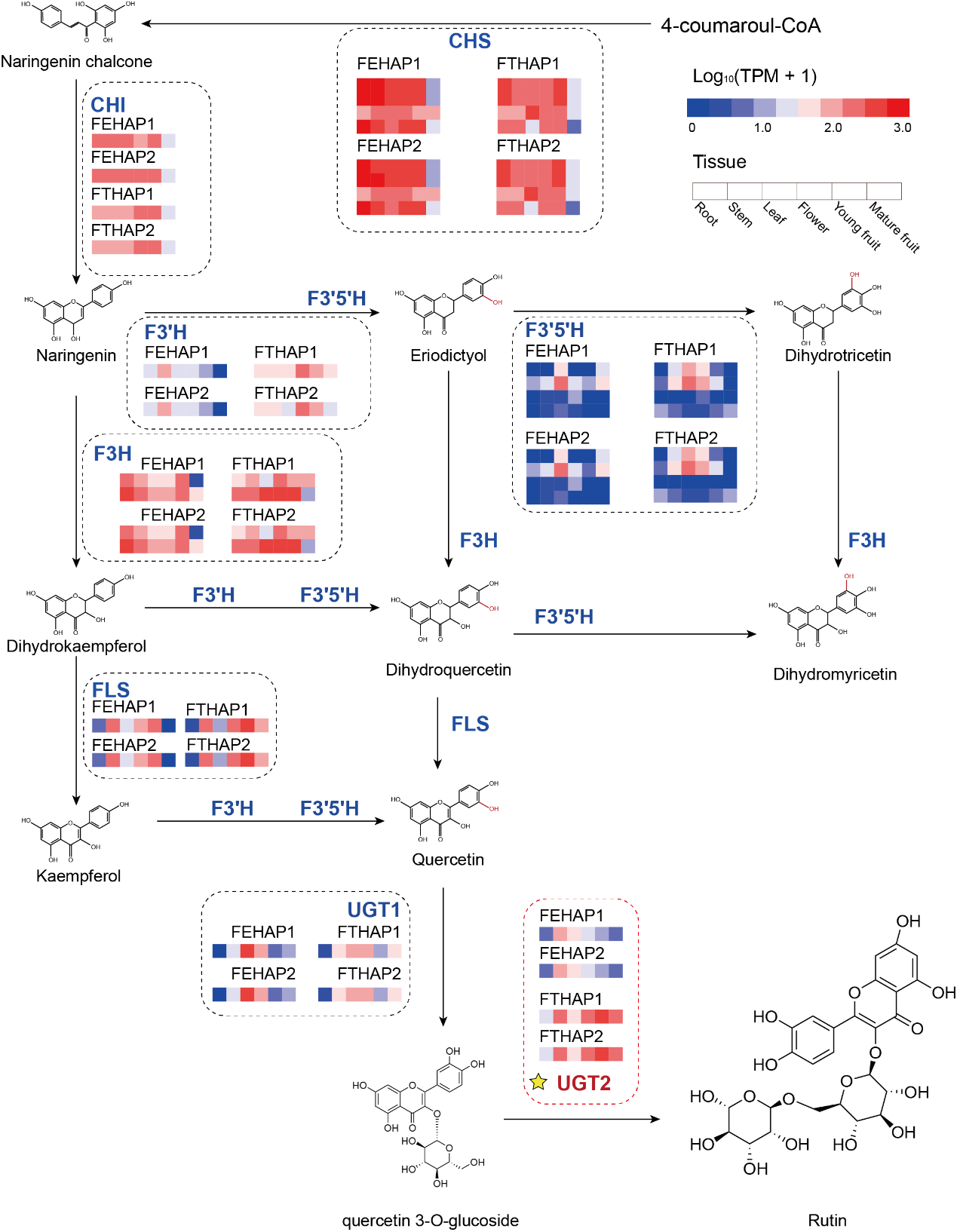


**Fig. S15** Overview of the rutin biosynthetic pathway in *F. tataricum* and *F. esculentum* with expression profiles of key enzyme genes.


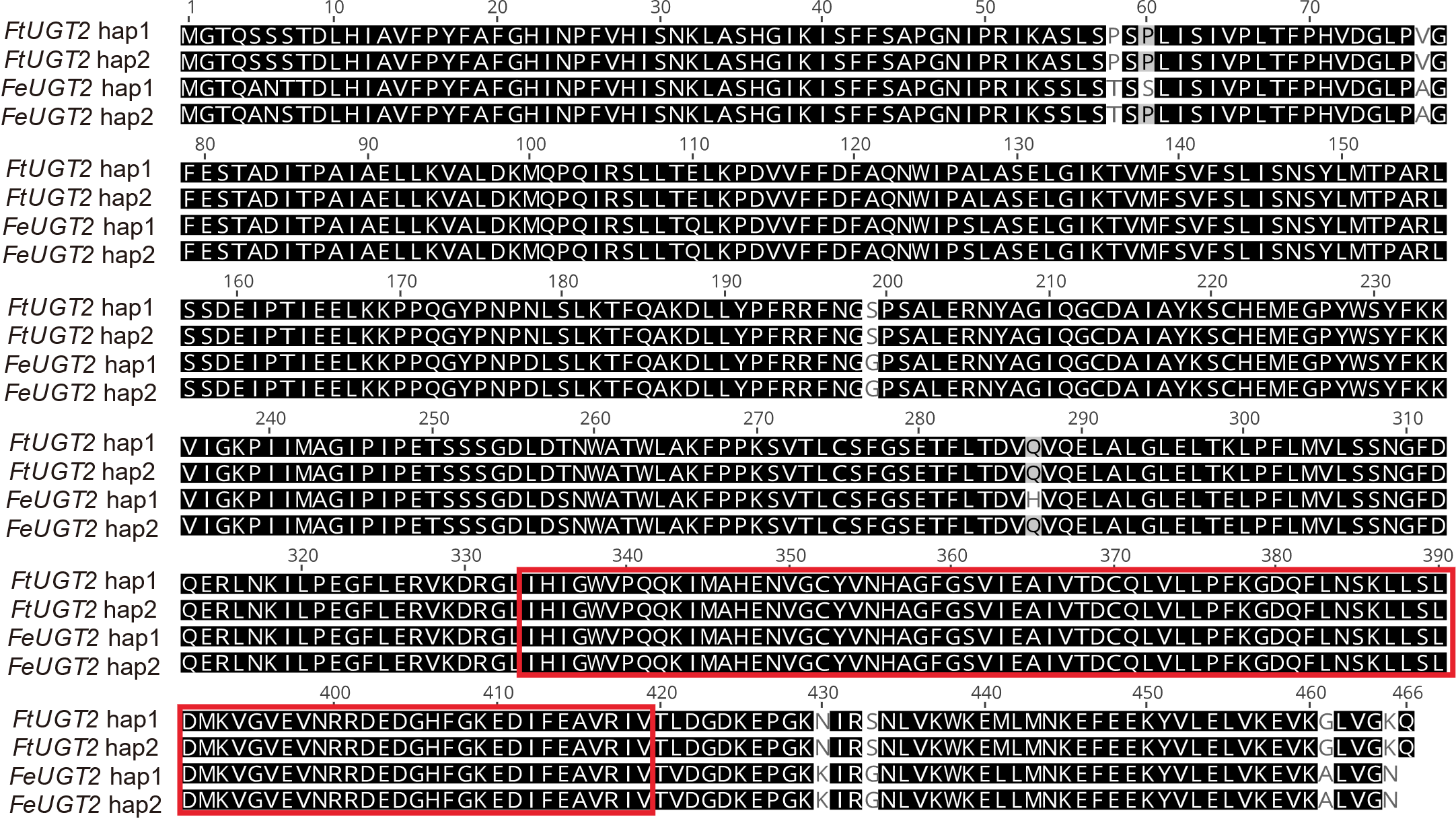


**Fig. S16** Multiple sequence alignment of the UGT2 proteins for the 4 assemblies. Red box indicates the position of UDP-glycosyltransferase functional domian (PF00201).


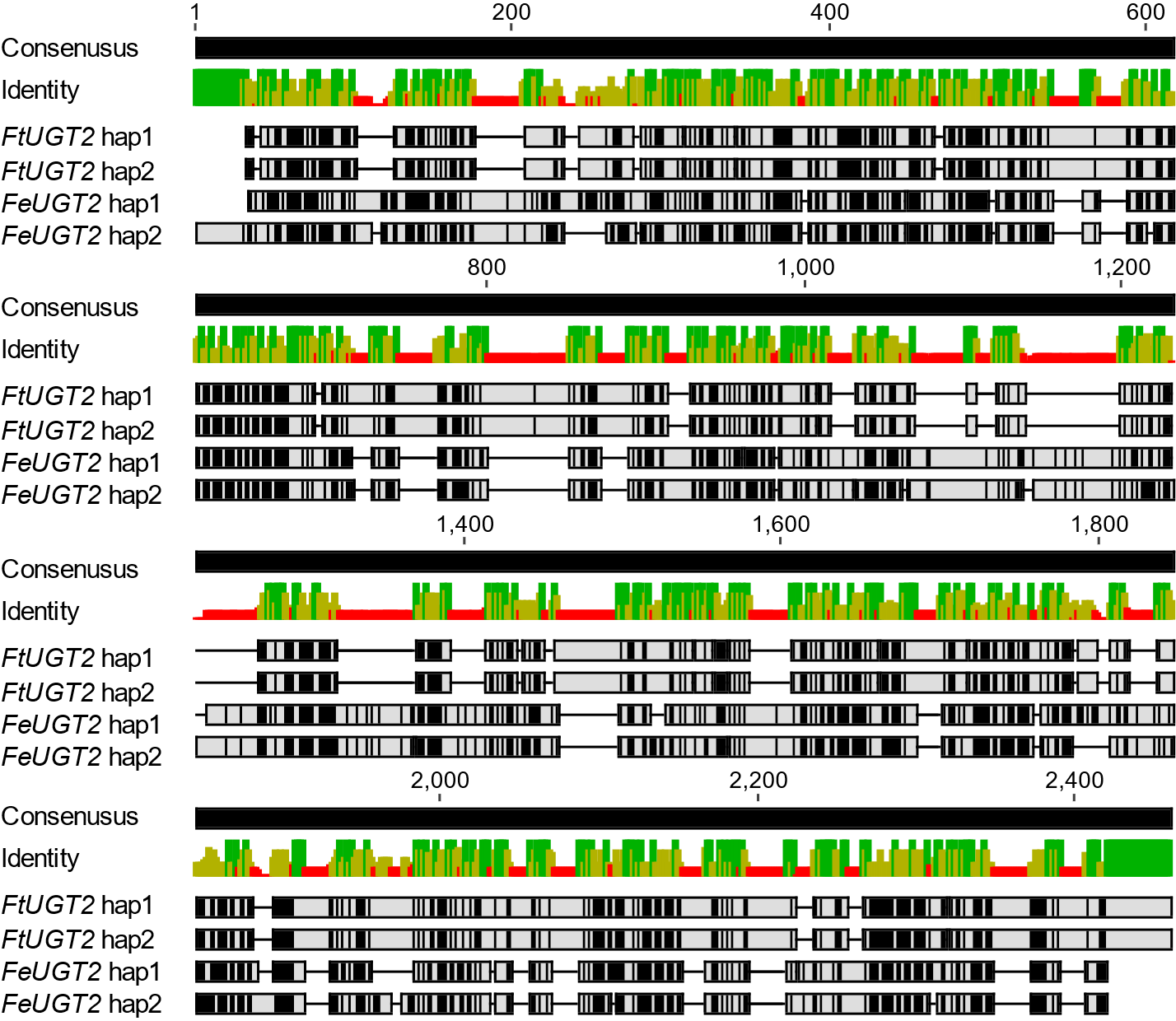


**Fig. S17** Sequence alignment of UGT2 promoter sequences in *F. tataricum* and *F. esculentum* haplotyped genomes.


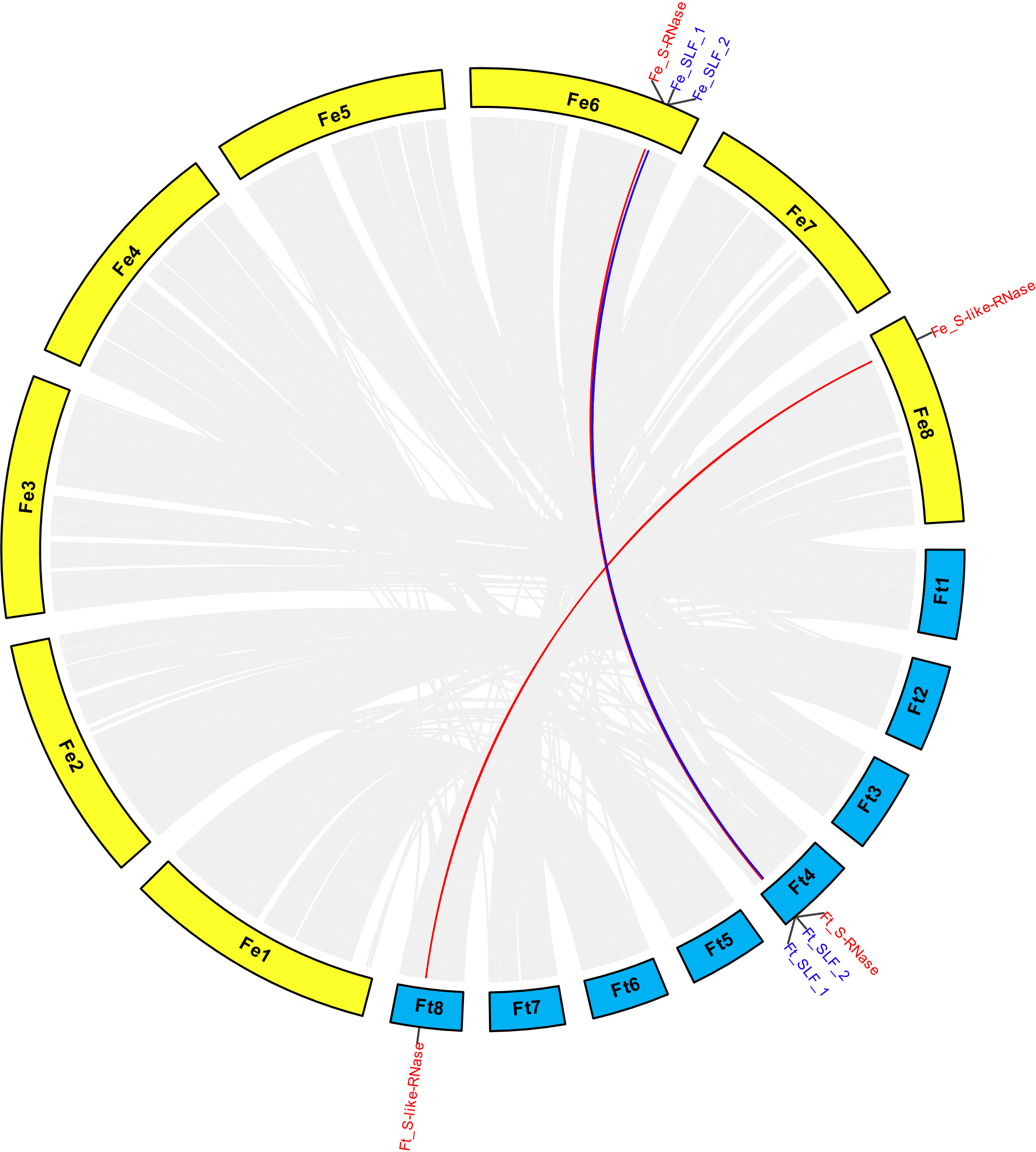


**Fig. S18** Gene collinear relationship between *F. tataricum* (n=8) and *F. esculentum* (n=8) genomes. Red lines indicate S-RNase genes loci while blue lines indicate SLF genes loci.


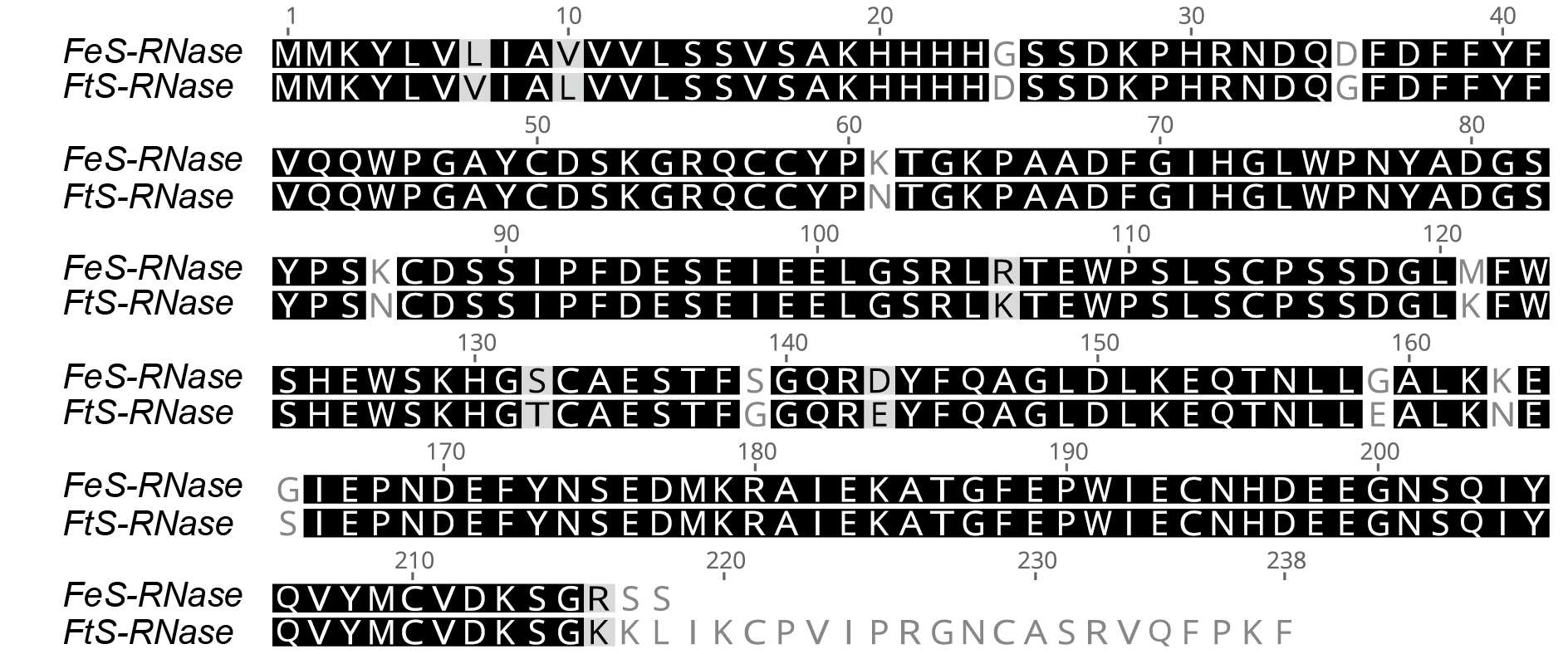


**Fig. S19** Multiple sequence alignment of the S-RNase proteins for the 2 assemblies


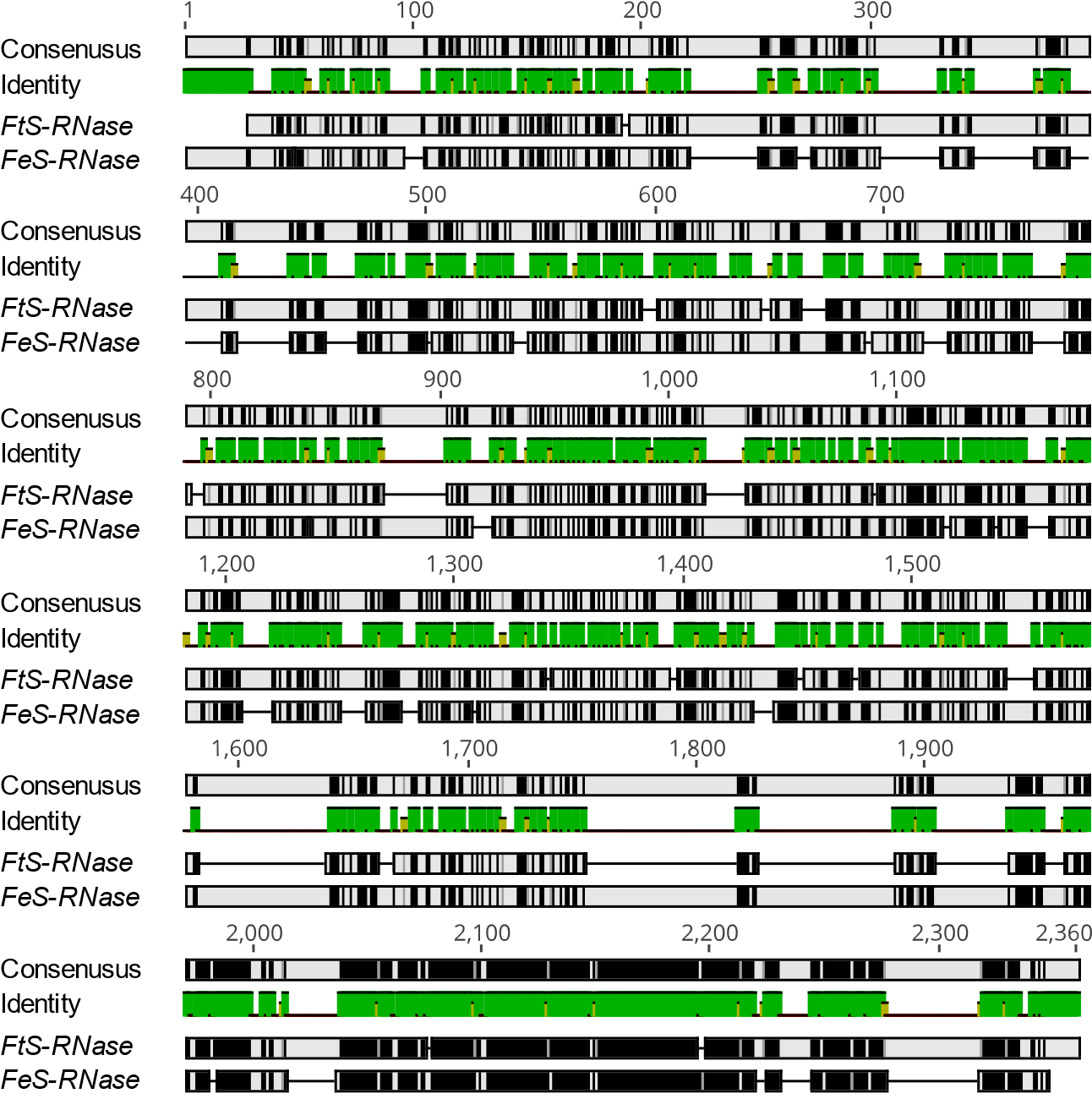


**Fig. S20** Sequence alignment of S-RNase promoter sequences in *F. tataricum* and *F. esculentum* genomes.


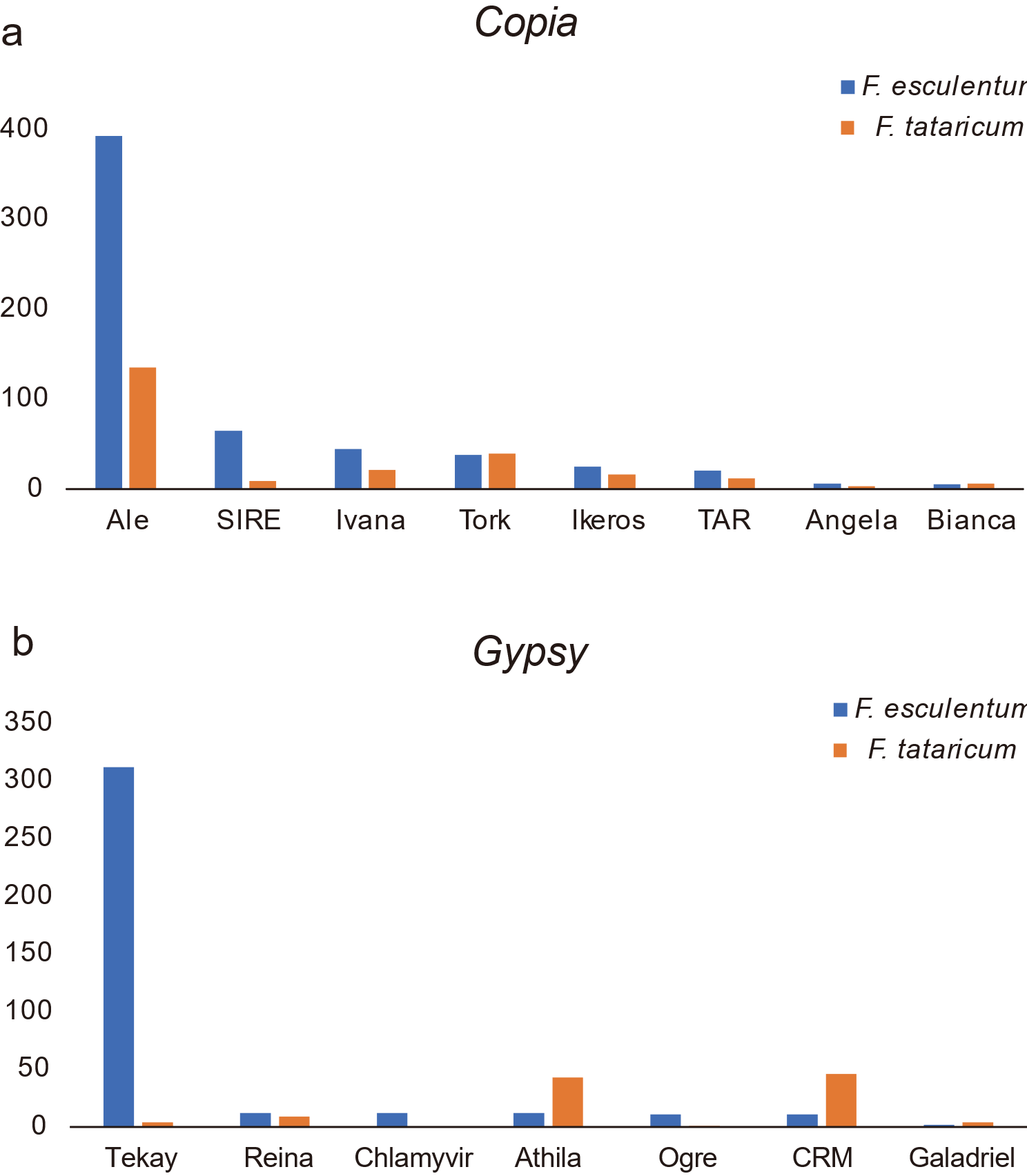


**Fig. S21** The number of different families within the *Copia* (a) and *Gypsy* (b) superfamilies.


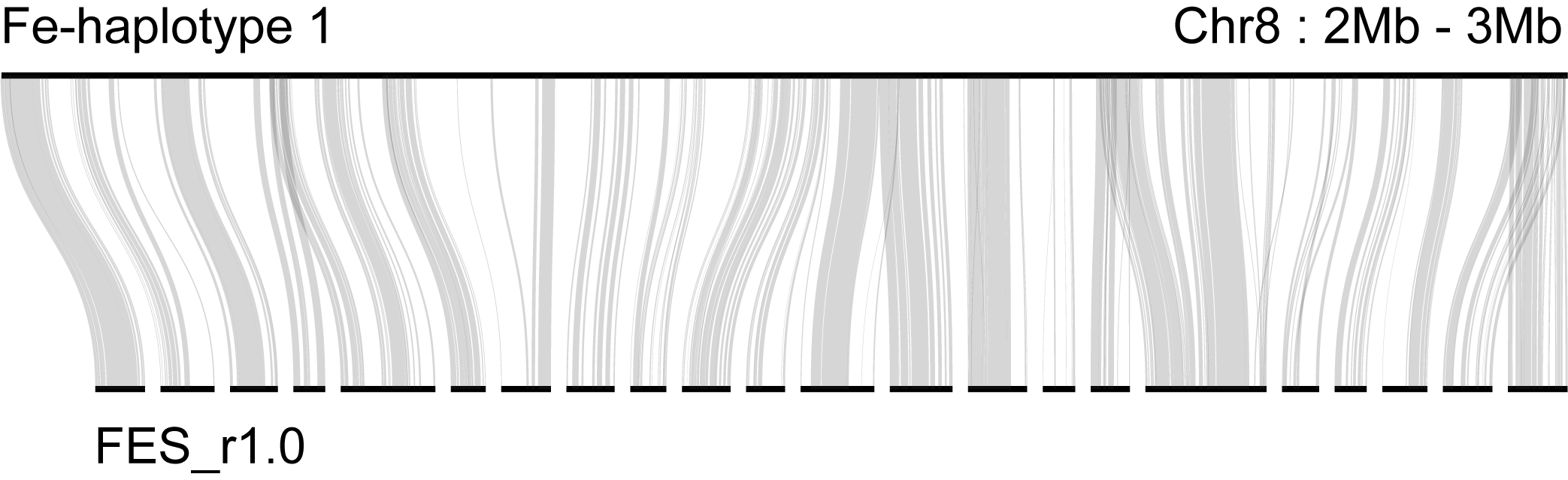


**Fig. S22** The genome comparison between the 2 Mb to 3 Mb interval of Chromosome 8 of Fe-haplotype 1 and FES_r1.0.
